# Computational profiling of hiPSC-derived heart organoids reveals chamber defects associated with *NKX2-5* deficiency

**DOI:** 10.1038/s42003-022-03346-4

**Published:** 2022-04-29

**Authors:** Wei Feng, Hannah Schriever, Shan Jiang, Abha Bais, Haodi Wu, Dennis Kostka, Guang Li

**Affiliations:** 1 Department of Developmental Biology, University of Pittsburgh School of Medicine, Pittsburgh, PA USA; 2 Joint Carnegie Mellon, University of Pittsburgh Ph.D. Program in Computational Biology, Pittsburgh, PA USA; 3 Vascular Medicine Institute Division of Cardiology, University of Pittsburgh Department of Medicine, Pittsburgh, PA USA; 4 Department of Computational & Systems Biology and Pittsburgh Center for Evolutionary Biology and Medicine, University of Pittsburgh School of Medicine, Pittsburgh, PA USA

**Keywords:** Heart development, Induced pluripotent stem cells

## Abstract

Heart organoids have the potential to generate primary heart-like anatomical structures and hold great promise as in vitro models for cardiac disease. However, their properties have not yet been fully studied, which hinders their wide spread application. Here we report the development of differentiation systems for ventricular and atrial heart organoids, enabling the study of heart diseases with chamber defects. We show that our systems generate chamber-specific organoids comprising of the major cardiac cell types, and we use single cell RNA sequencing together with sample multiplexing to characterize the cells we generate. To that end, we developed a machine learning label transfer approach leveraging cell type, chamber, and laterality annotations available for primary human fetal heart cells. We then used this model to analyze organoid cells from an isogeneic line carrying an Ebstein’s anomaly associated genetic variant in *NKX2-5*, and we successfully recapitulated the disease’s atrialized ventricular defects. In summary, we have established a workflow integrating heart organoids and computational analysis to model heart development in normal and disease states.

## Abstract (web summary)

A human cardiac organoid system, coupled with single cell RNA sequencing and machine learning for transcriptional phenotyping, was developed. This allowed investigation of a genetic variant associated with Ebstein’s Anomaly, a congenital heart disease with chamber defects.

## Introduction

Human induced pluripotent stem cells (hiPSCs) have been shown to differentiate into beating heart muscle cells (cardiomyocytes, CMs) with monolayer differentiation protocols, or into heart organoids comprised of a variety of cell types with three-dimensional differentiation systems^1–4^. While monolayer differentiation protocols are able to produce very pure populations of cells, they are not able to model the three-dimensional spatial microenvironments of cardiac development; therefore, these protocols may not be appropriate to study congenital heart defects (CHDs) in general. CHDs are the most frequently observed type of malformation at birth and the most common cause of infant death due to birth defects in the United States^5^. In contrast to monolayer cells, organoids are generated using three-dimensional differentiation methods, which enables them to develop anatomical context through self-assembly. This has already been leveraged to study developmental processes in several tissue and organ systems like brain, intestine, and kidney^6–8^. Also in the context of heart development several three-dimensional differentiation protocols have been published^3,9–13^, but their chamber identities have not been carefully investigated and applied to study CHDs. Therefore, we established two three-dimensional differentiation protocols geared towards producing atrial and ventricular heart organoids, respectively. This approach then allows us to study chamber defects in the context of CHDs in general and for Ebstein’s anomaly in particular.

Ebstein’s anomaly, a rare but serious CHD, occurs in ~1 in 200,000 live births and accounts for <1% of all cases of CHDs^14^. Patients suffer from heart chamber malformations, including enlarged right atrium (RA), reduced right ventricle (RV), and abnormal tricuspid valves. Genetic causes play a role in Ebstein’s anomaly, albeit the disease is genetically heterogeneous. Known genetic causes include chromosomal alterations (like copy number variations) and single gene defects in cellular structural proteins, signaling molecules, and cardiac transcription factors^15^. Specifically, multiple sequence variants within the homeobox-containing cardiac transcription factor *NKX2-5* have been associated with the disease^15,16^. Given that *NKX2-5* is a transcription factor with key roles in cardiac development^17–20^, and because knock out experiments in zebrafish and mouse have demonstrated that *NKX2-5* is involved in chamber specification in developing vertebrate hearts^21,22^, we were interested to further investigate a specific Ebstein’s anomaly-associated variant in the coding sequence of *NKX2-*5, where a cytosine is converted to an adenine (c.673C > A)^16^.

We used our differentiation system in combination with single-cell RNA sequencing (scRNA-seq) to address this question. scRNA-seq enables the study of transcriptional profiles of individual cells, and it has successfully been used to study and elucidate disease etiology for CHD^23–25^. Commercial droplet-based methods (like the 10X genomics platform) have been shown to capture a large diversity of cell types, and they can be used to assay a large number of cells in each experiment^26^. We utilized this approach to characterize organoids generated by our protocols at different differentiation time points, and it enabled us to compare wild-type heart organoids with organoids that were genetically modified to carry the *NKX2-5* c.673C > A variant. A major consideration in the design of scRNA-seq experiments are batch effects, which arise when samples are processed in separate groups. Batch effects have the potential to severely confound analysis results and downstream conclusions^26,27^. Therefore we used the MULTI-seq approach that (through lipid-based sample barcoding) enables multiplexing of different samples for library preparation and sequencing^28^.

For comprehensive molecular characterization of cardiac cells based on scRNA-seq, we used machine learning to implement a label transfer approach (based on random forests) that allowed us to leverage information about cell type, heart chamber (atrial vs. ventricular) and laterality (left vs. right side) available in primary human fetal cells^23,29,30^. The random forest learning algorithm is a machine learning method that has been successfully employed in the context of scRNA-seq data annotation^22^, and we adopted and modified this approach to generalize well across different sequencing platforms, and to include an anomaly detection step to highlight cells that are likely not heart-related. This enabled us to characterize the differentiation protocols we established and to compare wild type with genetically modified cells.

Overall, we find that our differentiation approach generates organoids containing heart cells with predominantly atrial or ventricular lineage identity, based on differentiation conditions. Single-cell transcriptional profiling in combination with the label transfer approach we developed was able to identify a range of cardiac cells in our organoids. Comparison of cells from wild-type organoids with cells from organoids with the Ebstein’s anomaly-associated genetic lesion *NKX2-5* c.673 C > A identified chamber developmental defects. Additionally, we found genes down-regulated in mutant cells are related to striated muscle differentiation, while up-regulated genes are related to energy and metabolism, illustrating specific molecular consequences of this genetic manipulation in the context of heart development. This finding suggests that our overall approach is a promising option for characterizing lineage defects and the functional roles of genetic variants in CHDs.

## Results

### Generation of ventricular-lineage heart organoids

**Fig. 1:**
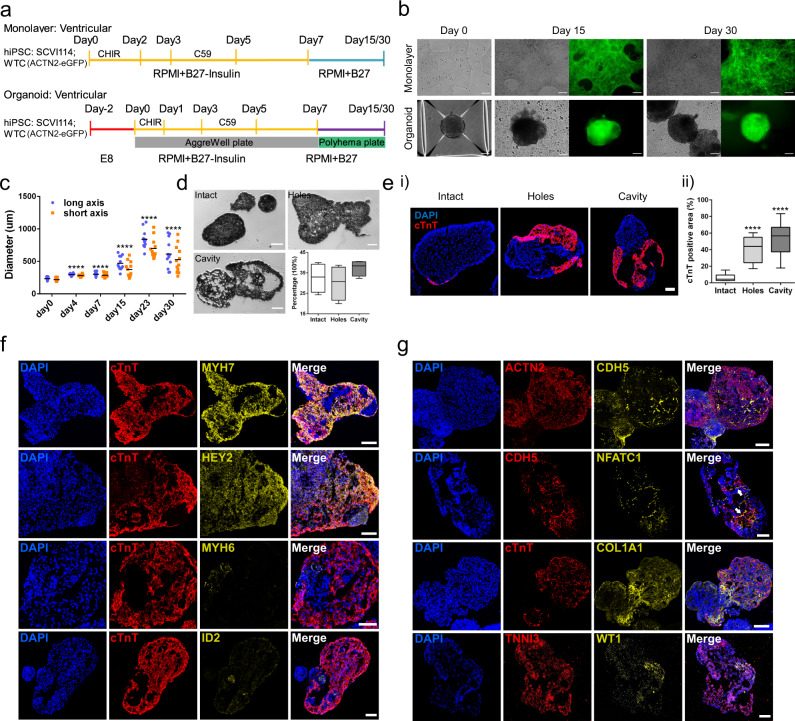
Differentiation and characterization of RA- (ventricular protocol) heart organoids. **a** Diagram of the RA- organoid and monolayer cell differentiation workflow. Two cell lines were differentiated in each system. **b** Representative images of the differentiated cells. The green signal represents CMs labeled by Actn2-eGFP. **c** Quantification of the organoid diameters from day 0 to day 30. *n* = 12 organoids in each group. ****p < 0.0001, Student’s *t* test against day 0. **d** Transverse section analysis revealed three types of organoids. Data are plotted as mean ± SEM, *n* = 4 biological independent RA- organoid differentiation. **e** Analysis of the CM areas in the three types of organoids based on cTnT staining. *N* = 13 intact organoids, *N* = 13 organoids with cavities, *N* = 11 organoids with holes. ****p < 0.0001, Student’s *t* test against intact. **f** RA- organoid from day 30 stained with ventricular and atrial markers. Scale bar = 100 μm. **g** In situ expression analysis of cardiac lineage genes with immunofluorescence and RNA in situ hybridizations. Arrowhead points to the CDH5 and NFATC1 positive cells aligning along the inner layer of the cavity. Scale bar = 100 μm.

In order to generate ventricular-lineage heart organoids, we established a three-dimensional differentiation protocol by sequentially modulating the *WNT* signaling pathway, which is largely similar to the established monolayer differentiation protocols^2,10,31–33^. This allowed us to differentiate two hiPSC lines (WTC line with ACTN2-eGFP reporter and SCVI114 line) into cardiac lineages in organoid (Org) and monolayer (ML) systems (Fig. 1a). Beating cells and ACTN2-eGFP signal were observable at day 15 and 30 in both protocols (Fig. 1b, Supplementary Fig. 1a, Supplementary Video 1). Cardiomyocyte percentages in organoids were quantified with flow cytometry, using ACTN2-eGFP expression (Supplementary Fig. 1b). Interestingly, shorter sarcomere lengths, but not beating rates, were observed in organoid cells compared to monolayer cells (Supplementary Fig. 2a, c). Organoid size increased throughout early stages of differentiation and remained stable between day 15 and 30, but the variance increased markedly after day 7 (Fig. 1c) when cells had been transferred from AggreWell to six-well plates. Transverse sectioning of the organoids revealed varied internal structures, which we grouped into three categories: intact, holes, and cavities (Fig. 1d and Supplementary Fig. 3a). When staining with cardiac troponin T (cTnT), a marker for cardiomyocytes, we found that 90.9% of organoids with cavities and 71.4% of organoids with hole structures contained cTnT positive cells, while most organoids with intact structures (75%) were cTnT negative (Supplementary Fig. 3b, d). Quantification of cTnT-positive areas confirmed that organoids with cavities had the strongest cTnT signal followed by organoids with holes, while organoids with intact structures showed little signal (Fig. 1e). Furthermore, organoids with cavities and hole structures were found to be larger than the intact organoids, while cell numbers did not show significant differences (Supplementary Fig. 3e).

Ventricular identity of organoid cells was verified by marker gene examination. Immunostaining of ventricular markers MYH7 and HEY2^29,34,35^ showed a strong presence, while atrial markers MYH6 and ID2^29,36,37^ were identified in very few cTnT positive cells (Fig. 1f). These results suggest that most of the generated cardiomyocytes (CMs) with this protocol are indeed ventricular CMs.

Simultaneous staining of CDH5 and NFATC1 revealed a small number of endothelial cells lining the inner cell layer of the cavities (Fig. 1g), a similar pattern to what endocardial endothelial cells show in vivo^38^. Fibroblasts were found to either exist throughout the entire organoid or be located separately from cardiomyocytes based on their expression of COL1A1. Finally, RNA staining of *WT1* and *TNNI3* revealed the existence of epicardial cells in the organoids (Fig. 1g). Notably *WT1* expression was observed only in a small portion of cells, suggesting that epicardial cell or epicardial cell-derived cells only developed in a small region of the organoids.

Overall, these results show the heart organoids we generated with this protocol contained predominantly ventricular lineage cells and captured several important heart developmental characteristics observed in vivo; this implies their potential utility in studying ventricular cardiogenesis in vitro.

### Transcriptional analysis of ventricular-lineage organoids

**Fig. 2:**
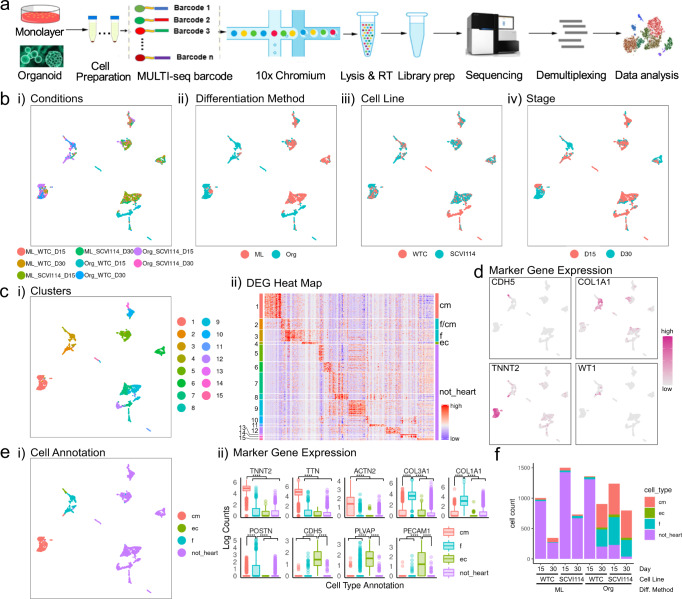
ScRNA-seq analysis of RA- (ventricular protocol) cells. **a** Diagram of the MULTI-seq experimental workflow. **b** UMAP projections of the single cells grouped by (i) conditions, (ii) differentiation methods, (iii) cell lines, and (iv) stages. As for all UMAPs in this work, the x-axis is UMAP1 and the y-axis is UMAP2. **c** Unsupervised clustering of the single cells. (i) UMAP projection of the clusters and (ii) cluster-specific differentially expressed genes with cluster labels (left) and annotated cell types (right). Annotations to each cluster available in a Source data file. **d** UMAP projections of single cells colored by the expression pattern of representative cardiac lineage genes. **e** (i) UMAP projection of single cells grouped by cell type and (ii) expression levels of lineage genes in each annotated cell type. *N* = 1519 CMs, 1143 fibroblasts, 125 endothelial cells, 5094 not_heart cells. ****p ≤ 0.0001, Bonferroni corrected *p* values from Wilcox test, exact *p*-values and sample numbers available in a Source data file. **f** The number of profiled cells in each condition colored proportionately by annotated cell type.

With the goal of better understanding the cellular and molecular heterogeneity of organoids generated by our protocol, we used single-cell RNA sequencing (scRNA-seq) to profile and analyze cells’ transcriptomes. To control for potential batch effects, we employed the MULTI-seq protocol^28^. In this approach each sample was pre-stained with a unique MULTI-seq sample barcode, and subsequently samples were pooled together and processed with the regular 10X single cell-profiling workflow with minor adaptions^39^. After Illumina sequencing, data was demultiplexed based on their MULTI-seq barcodes to identify sequencing reads from individual samples (Fig. 2a and Supplementary Fig. 4).

Using this approach, we profiled organoid and monolayer differentiated cells (WTC and SCVI114 cell lines) that were generated as described above. After read mapping, demultiplexing, and quality control (see Methods section, Supplementary Figs. 4 and 5), we recovered 3612 cells for the WTC cell line (2361 at day 15 and 1251 at day 30) and 4269 cells for the SCVI114 cell line (2740 at day 15 and 1529 at day 30) for further analysis. Unsupervised clustering analysis revealed major transcriptional differences between cells based on specific combinations of cell line, differentiation protocol (Org = organoid vs. ML = monolayer) and stage (day 15 vs. day 30) (Fig. 2b). We used graph-based clustering to cluster the cells into 15 distinct groups and identified corresponding unique gene expression signatures for each cluster (Fig. 2c and Supplementary Data 1). Together with expression of lineage marker genes (cardiomyocytes: *TNNT2*, *TTN*, *ACTN2*; endothelial cells: *CDH5*, *PECAM1*, *FLVAP*; fibroblasts: *COL1A1*, *POSTN*), we identified these three major cardiac cell types (Fig. 2d, e); non-heart-cells did not express cardiac lineage genes (Fig. 2eii). We found that D15 and D30 organoid cells of the SCVI114 cell line and D30 organoids cells from WTC cell line predominantly differentiated into cardiac cells with only a small percentage specified into non-heart cells; however, monolayer cells and D15 organoid cells from the WTC cell line mostly differentiated into non-heart cells (Fig. 2f). This observation may be the result of variation in CM differentiation efficiency between experiments. To assess monolayer CMs from low-efficiency samples we compared them to monolayer CMs from other research groups where scRNA-seq data has been reported in the literature^40,41^. We find that our CMs are transcriptionally most similar to CMs generated with these other protocols (Supplementary Tables 1 and 2), an observation suggesting CMs from low efficiency samples fall within the range of results typical for this type of setup. Finally, we also profiled CMs and non-CMs enriched by FACS based on ACTN2-eGFP expression and identified similar results for monolayer cells at day 15 (Supplementary Figs. 6 and 9e). Overall, scRNA-seq analysis confirmed prior observations about cell function and morphology and showed we were able to generate organoids predominantly consisting of cardiac cell types (cardiomyocytes, fibroblasts, endothelial cells).

### Generation of atrial-lineage heart organoids

**Fig. 3:**
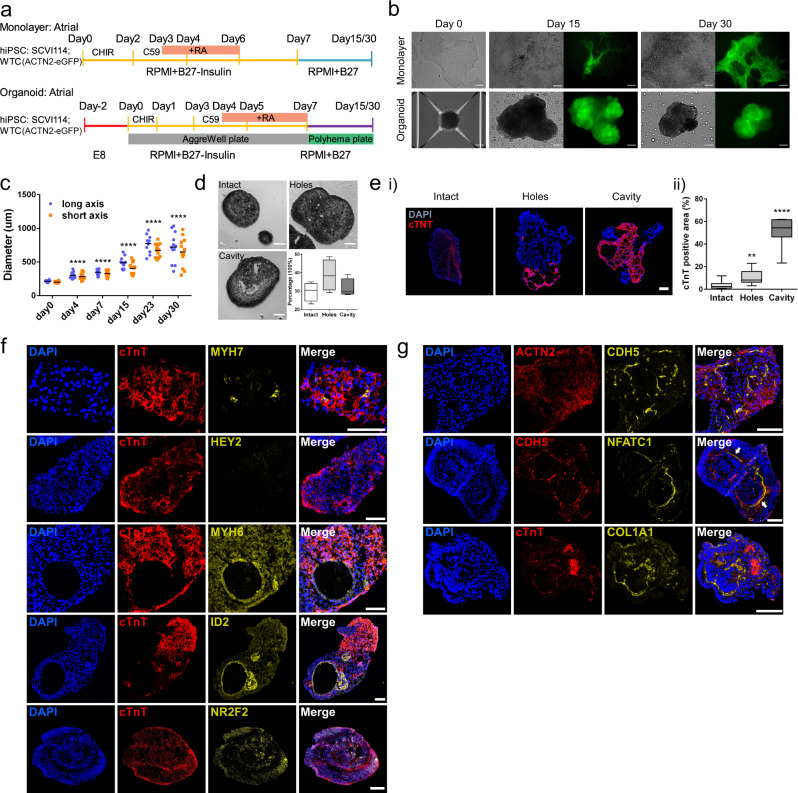
Differentiation and characterization of RA+ (atrial protocol) heart organoids. **a** Diagram of the RA+ organoid and monolayer cell differentiation workflow. RA was added to induce the atrial cell lineages. **b** Representative image of the cells at different differentiation stages. The green signal represents CMs labeled by Actn2-eGFP. **c** The diameter of RA+ organoids from day 0 to day 30. *n* = 12 organoids in each group. ****p < 0.0001, Student’s *t* test against day 0. **d** Three types of atrial organoids were identified based on their internal structures. Data are plotted as mean ± SEM, *n* = 4 biological independent RA+ organoid differentiation. **e** Analysis of the CM areas in the three types of atrial organoids based on cTnT staining. *n* = 14 organoids in intact, *n* = 10 organoids in holes, *n* = 11 organoids in cavity. **p < 0.01, ****p < 0.0001, Student’s *t* test against intact. **f** Immunofluorescence analysis of atrial and ventricular marker genes expression at day 30 RA+ organoids. Scale bar = 100 μm. **g** Immunofluorescence staining analysis of cardiac lineage genes in RA+ organoids. Arrowhead points to the CDH5 and NFATC1 positive cells aligning along the inner layer of the cavity. Scale bar = 100 μm.

In order to generate atrial-lineage heart organoids, we modified our previous differentiation workflow by treating cells with retinoic acid (RA) at cardiac mesoderm and progenitor stages, similar to monolayer atrial differentiation^42^ (Fig. 3a). Moving forward, we use RA+ to refer to atrial lineage heart organoids and RA- to refer to ventricular lineage heart organoids. In line with RA- organoids, we differentiated WTC and SCVI114 cell lines and observed beating cells and ACTN-eGFP signal at day 15 and day 30 (Fig. 3b, Supplementary Fig. 1a, and Supplementary Video 2). As before, we quantified beating rates and found that RA+ organoids showed significantly lower beating rates compared to monolayer cells (Supplementary Fig. 2b). Similar to RA- organoids, we found that RA+ organoids grew fast at early stages, then between day 15 and 30 the average size remained similar, but the variance increased markedly (Fig. 3c). Again, transverse section analysis of the organoids identified three types of internal structures, intact, hole, and cavity (Fig. 3d and Supplementary Fig. 3a). Staining for cTnT revealed that ~66.7% of organoids with cavities, ~23.3% of organoids with holes, and ~15.4% of organoids with intact structures contain cTnT positive cells (Supplementary Fig. 3c, d). Quantification of cTnT positive areas further confirmed that organoids with cavities had the largest CM areas on average (Fig. 3e).

Immunostaining analysis of RA+ organoids showed high levels of the atrial markers MYH6, ID2, and NR2F2^22,29,37,43^, but low levels of ventricular markers MYH7 and HEY2^29,34,35^, suggesting this protocol produced predominantly atrial CMs (Fig. 3f). Furthermore, co-staining of CDH5 and NFATC1 identified a large proportion of ECs lining the interior of the cavities, and the staining of COL1A1 found fibroblasts developed throughout the entire organoid (Fig. 3g). Percentages of these two cell types were further quantified by FACS analysis of CDH5 and COL1A1 expression (Supplementary Fig. 7a, b). Additionally, we found the organoids with cavities and holes had higher EC percentages than the organoids with intact structures, while fibroblasts percentages were not significantly different (Supplementary Fig. 7c, d).

Overall, similar to RA- organoids, we have developed a protocol to generate organoids with predominantly atrial lineage cells and the potential to become a valuable in-vitro tool to study atrial cardiac lineage development.

### Transcriptional analysis of atrial-lineage organoids

**Fig. 4:**
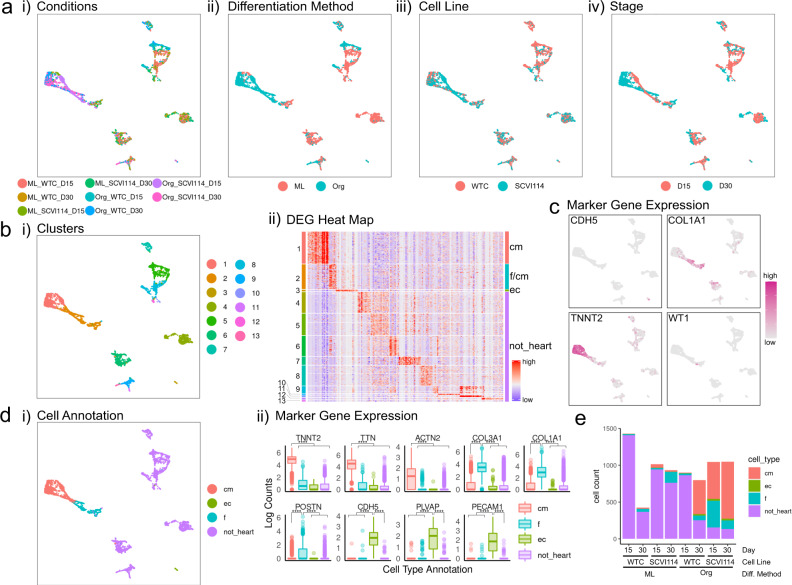
ScRNA-seq analysis of RA+ (atrial protocol) cells. **a** UMAP projections of the single cells grouped by (i) conditions, (ii) differentiation methods, (iii) cell lines, and (iv) stages. **b** Unsupervised clustering of the single cells. (i) UMAP projection of the clusters and (ii) cluster-specific differentially expressed genes with cluster labels (left) and annotated cell types (right). Annotations to each cluster available in a Source data file. **c** UMAP projections of single cells colored by the expression pattern of representative cardiac lineage genes. **d** (i) UMAP projection of single cells grouped by cell type and (ii) expression levels of lineage genes in each annotated cell type. *N* = 1836 CMs, 789 fibroblasts, 82 endothelial cells, 4886 not_heart cells. ****p ≤ 0.0001, Bonferroni corrected *p* values from Wilcox test, exact *p*-values and sample numbers available in a Source data file. **e** The number of profiled cells in each condition colored proportionately by annotated cell type.

Within the same MULTI-seq experiment as for RA- organoids, we also profiled cells from RA+ organoid and monolayer differentiations. Differentiated cells at D15 and D30 from WTC and SCVI114 cell lines were analyzed as described above. We recovered 3551 cells for the WTC cell line (2329 at day 15, 1222 at day 30) and 4042 cells for the SCVI114 cell line (2060 at day 15 and 1982 at day 30) for further analysis. Unsupervised clustering followed by projection into two dimensions revealed clear transcriptional differences between differentiation protocols (Org vs. ML) and stages (D15 vs. D30), while differences between the two cell lines (WTC vs. SCVI114) were more subtle and most pronounced in non-heart cells (Fig. 4ai–iv). Cells were grouped into 13 clusters using graph-based clustering and we identified unique expression signatures in each of them (Fig. 4bi, ii and Supplementary Data 2). Again, making use of lineage marker genes, we identified CMs, endothelial cells, fibroblasts, and non-heart cells (Fig. 4c, d). Consistent with what we observed in RA- organoids, we found that most SCVI114 organoid cells (D15 and D30) and most cells from WTC organoids at D30 differentiated into cardiac cells, whereas WTC and monolayer cells at D15 mainly comprise “non-heart” cells (Fig. 4e). Finally, we also profiled RA+ CMs (D15 and D30) enriched by FACS based on ACTN2-eGFP expression and found they were a highly pure population of CMs (Supplementary Fig. 8a–c and Supplementary Data 3). Overall, scRNA-seq analysis confirmed prior observations and showed we were able to generate organoids predominantly consisting of cardiac cell types (CMs, fibroblasts, endothelial cells) with the majority of CMs having atrial-lineage.

### Comparative analysis of RA- and RA+ organoids

**Fig. 5:**
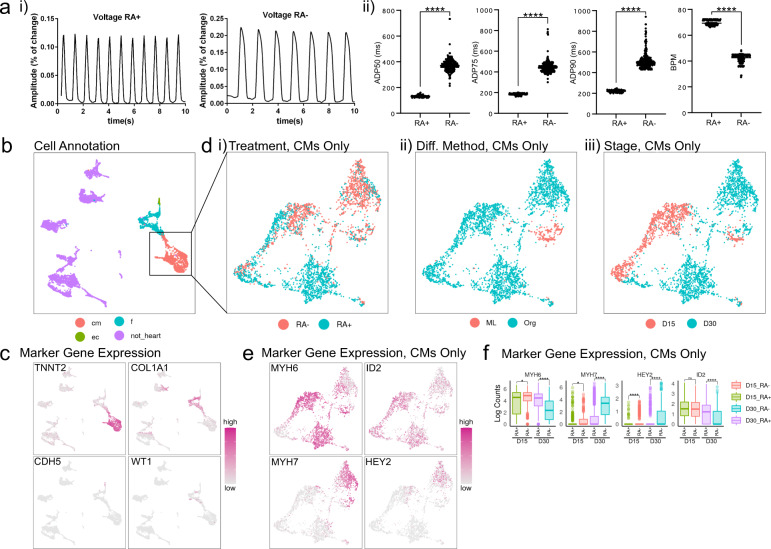
Co-analysis of RA- and RA+ cells. **a** (i) Representative plots of membrane potential in RA- and RA+ organoid derived CMs at Day 30 and (ii) quantifications of action potential duration (APD) and beating rates in the cells. For APD, *n* = 299 cells in RA-, *N* = 129 cells in RA+. For BPM, *n* = 298 cells in RA-, *N* = 124 cells in RA+. ****p < 0.0001, Student’s *t* test. **b** UMAP projection of single cells grouped by cell type. **c** UMAP projections of single cells colored by the expression pattern of representative cardiac lineage genes. **d** UMAP projection of CMs grouped by (i) treatments, (ii) differentiation methods, and (iii) stages. **e** UMAP projections of CMs colored by the expression pattern of atrial and ventricular marker genes. **f** Expression levels of atrial and ventricular marker genes in each conditioned CMs. *N* = 592 D15_RA-, 563 D15_RA+, 927 D30_RA-, 1273 D30_RA+. ns *p* > 0.05, *p < = 0.05, ****p < = 0.0001, Bonferroni corrected *p* values from Wilcox test, exact *p*-values and sample numbers available in a Source data file.

Membrane potential analysis revealed typical atrial and ventricular action potentials in the RA+ and RA- organoid derived CMs, respectively (Fig. 5ai). Further quantification of the recordings identified shorter action potential duration (APD) 50, APD75, APD90, and higher beating rates in the RA+ organoid cells than RA- organoid cells (Fig. 5aii). Meanwhile, co-analysis of the scRNA-seq data from the two differentiation methods revealed three major cardiac cell types and non-heart cells (Fig. 5b, c). Within CMs, RA- and RA+ organoid-derived CMs were largely transcriptionally distinct at day 30 but not at day 15 on UMAP plots (Fig. 5d), suggesting the CMs have stronger chamber identities at day 30. This was further confirmed by expression of the marker genes MYH6, ID2 (atrial markers) and MYH7, HEY2 (ventricular markers) in RA+ and RA- CMs at day 30 (Fig. 5e, f).

These results imply that our two differentiation protocols generated the desired atrial and ventricular-lineage organoids, enabling us to compare and contrast atrial and ventricular differentiations in normal and pathological conditions.

### Iterative application of random forests for label transfer from human fetal heart cells

**Fig. 6:**
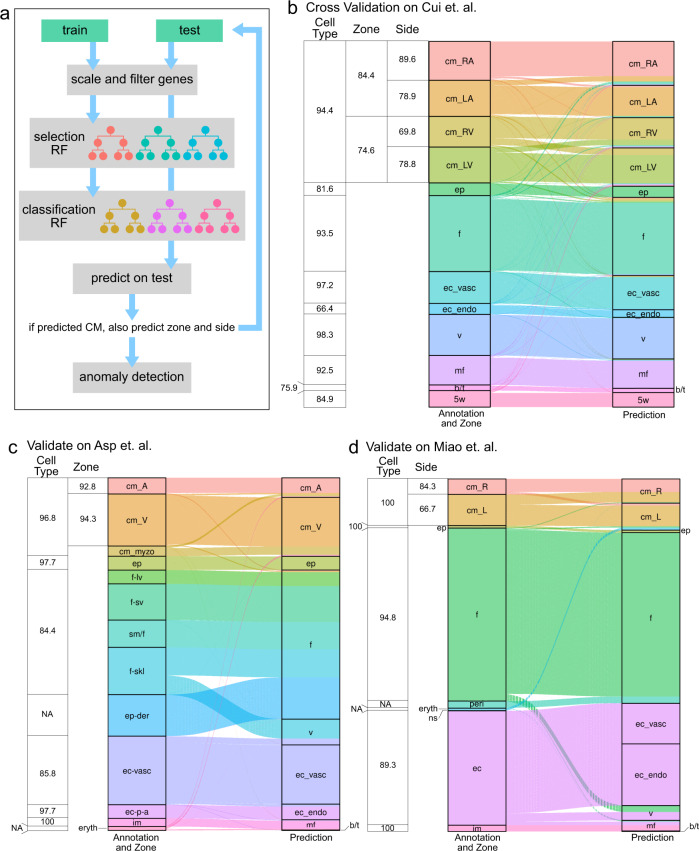
Predicting cardiac cell types, anatomical zones, and laterality. **a** Diagram of hierarchal random forest model. Train and test data are aligned via scaling. Train data is used to derive a random forest model for cell type, which is then applied to test data. For cardiomyocytes the procedure is iterated for predicting anatomical zone and laterality. After prediction, anomaly detection removes cells types from the test dataset that were not present in the training dataset. **b** Sankey diagram of 10-fold cross-validation results on data from Cui et al.^29^. Table provides cell type, conditional zone, and conditional laterality/side accuracies. **c** Sankey diagram and accuracies of prediction results for training on the Cui et al. data and prediction on the Asp et al data^30^. **d** Sankey diagram and conditional accuracies of prediction results on Miao et al. data^23^ (training again on the Cui et al. data). cm cardiomyocytes, ep epicardial cell, f fibroblast, ec_vasc vascular endothelial cells, ec_endo endocardial endothelial cells, v valvar, mf macrophage, b/t b/t cell, 5w undifferentiated cells, cm_myzo Myoz2-enriched CMs, f-lv fibroblast-like: (related to larger vascular development), f-sv fibroblast-like: (related to smaller vascular development), sm/f smooth muscle cells/fibroblast-like), f-skl fibroblast-like: (related to cardiac skeleton connective tissue), ep-der epicardium-derived cells, ec-p-a endothelium/pericytes/adventitia, im immune cells, eryth erythrocytes, peri pericyte, ns nervous system, LA left atrium, RA right atrium, LA left ventricle, RV right ventricle.

In order to more objectively characterize scRNA-seq data generated from our organoids, we developed a computational approach based on the random forest classification algorithm^44^. Our goal was to annotate cells from our organoids using published information about (cardiac) cell type, anatomical zone (ventricular vs. atrial), and laterality (left vs. right) from human fetal cells in Cui et al.^29^ Briefly, to transfer cell type labels we used the Cui et al dataset to train a feature selector random forest and a classifier random forest that can then be applied to predict the cell type in test data (our organoid cells, for example). Cells predicted as CMs can then optionally be classified further to label anatomical zone and laterality. Finally, our method can perform anomaly detection to filter out cell types that were not present in training data (Fig. 6a and Supplementary Fig. 9). This approach also enables us to focus on cardiac cells in our comparisons.

We assessed the performance of this approach in three ways: First, we performed 10-fold cross validation on the Cui et al. data itself. While cross validation guards against overfitting, this is an optimistic scenario because it does not take into account potential differences between training and test datasets. We found that in this setting our approach accurately predicted cell types, anatomical zones and lateralities (Fig. 6b). We noted, though, that performance for cell type prediction worked better (average accuracy = 87.19%) than predicting anatomical zone or laterality (average conditional accuracies of 81.5 and 79.28%, respectively). Second, to take platform differences, variations between laboratories, and other biological variables into account, we used the trained model to predict on data from Asp et al.^30^, which contained cell type and zone labels and was profiled with the 10X platform (Cui et al. used STRT-seq) (Fig. 6c). Again, we observed highly accurate prediction of cardiac cell types (average accuracy = 93.73%) and anatomical zones (average conditional accuracy = 93.55%). Interestingly, epicardial derived cells (ep_der), which mostly are fibroblasts, were correctly predicted as such; cardiac skeleton like fibroblasts, however, were predicted as valve cells. Third, we used the trained model to predict on 10X data from Miao et al.^23^ to assess cell type prediction a second time and, importantly, to assess cross platform laterality prediction. Consistent with our previous results we found cell type prediction highly accurate (average accuracy = 96.82%); prediction of laterality however was only moderately successful, with ~84% of CMs on the right side and ~66% of CMs on the left side correctly classified (Fig. 6d), with many left-annotated CMs misclassified as right.

Overall, these results showed that we can use the Cui et al. data set to annotate scRNA-seq data generated on different platforms by different laboratories. We conclude that we can be highly confident in cell type annotations, confident anatomical zone annotations, and moderately confident in laterality annotations.

### Computational annotation of heart organoid cells’ transcriptomes

**Fig. 7:**
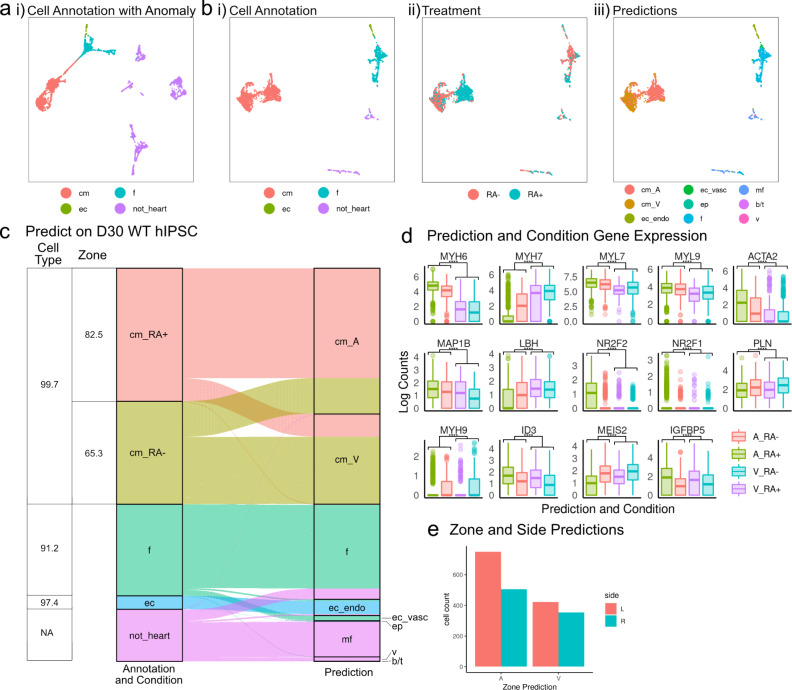
Prediction of wild-type RA- and RA+ cells using the validated hierarchal random forest model. **a** UMAP projections of RA- and RA+ single cells at day 30 grouped by cell type annotation. **b** UMAP projections of wild-type RA- and RA+ single cells at day 30 with Anomalies removed grouped by (i) cell types, (ii) treatment, and (iii) predicted cell types and zone. **c** Sankey diagram of prediction results of wild-type RA- and RA+ cells at day 30 (Anomalies removed). Table provides cell type and conditional zone accuracies. **d** Expression levels of genes used to make prediction decisions that either correlate with the predictions (A and V) or experimental conditions (RA- and RA+). ****p ≤ 0.0001, Bonferroni corrected *p* values from Wilcox test, exact *p*-values and sample numbers available in a Source data file. *N* = 308 A_RA-, 580 V_RA-, 947 A_RA+, 195 V_RA+. **e** Bar plot of side predictions in WT cells at day 30.

Next, we used our computational approach to annotate RA+ and RA- cells at day 30 (Fig. 7). We found that anomaly detection mainly removed non-heart cells as expected, and of the few heart cells that were filtered out, most were intermediate cells between fibroblasts and cardiomyocytes (Supplementary Fig. 9c). After removal of anomalous cells, cardiac cell types (CMs, fibroblasts, endothelial cells) account for the vast majority of cells and contributed to the major variations in this data set (Fig. 7b). This allowed us to exclusively focus on cardiac cell types for downstream analysis. Furthermore, remaining non-filtered non-heart cells were predicted as immune cells (macrophages, b/t cells) and fibroblasts. Visual inspection showed that global transcriptional differences between RA- and RA+ differentiation protocols are most strongly apparent in CMs (Fig. 7bii), a trend that was also captured by our anatomical zone predictions (Fig. 7biii). Cell type predictions are 91% consistent with our manual cell type annotations, which is also consistent with expectations derived from the validation results as described above. Consistent with the marker gene staining characterization of RA+ organoids presented above, classification results for RA+ cardiomyocytes are predominantly “atrial” (82.5%). For RA- cardiomyocytes, however, we find lower fraction of cells classified as “ventricular” (65.3%), and therefore a minority but sizeable fraction of 34.7% of RA- cardiomyocytes are classified as “atrial” (Fig. 7c). Furthermore, analyzing CMs in the context of anatomical zone prediction (Fig. 7d) we found genes that lend support to computational zone predictions (e.g*., MYL7* and *MYH6* (atrial markers)*, MYH7* (ventricular marker)), while others were more consistent with differentiation protocols (*PLN*, *MYH9*, and *MEIS2* (ventricular markers) and *ID3* and *IGFBP5* (atrial markers)^37^). Other marker genes (*NR2F1, NR2F2*)^22,42,43^ were less clear to interpret. In terms of laterality prediction, we observed that more “left” than “right” CMs, and we found this bias more pronounced for predicted “atrial” CMs compared with predicted “ventricular” CMs (Fig. 7e). For cells from day 15 organoids we found mostly consistent results (Supplementary Fig. 10; cell type predictions were highly accurate (average accuracy = 95.53%), RA+ CMs were largely predicted as “atrial” (91.2%), however a large fraction of RA- CMs (84.6%) were also predicted as “atrial”. Also, most CMs at day 15 were not substantially different between RA+ and RA- differentiation protocols (Supplementary Fig. 10aiv, b), indicating CMs at day 15 may not have matured enough to gain zone identities, which was further supported by the detailed comparative analysis of cells at day 15 and 30 (Supplementary Fig. 11 and Supplementary Data 4).

We also analyzed the cardiac cells enriched by FACS. We again found cell type predictions highly accurate (average accuracy = 94.4%), as were anatomical zone predictions: 94.7% of RA+ CMs (atrial protocol) were predicted as atrial CMs, and 62.9% of RA- CMs (ventricular protocol) were predicted as ventricular CMs (Supplementary Fig. 8d).

Overall, our automatic predictions achieved high accuracy in cell type annotations and highlighted that zone identities were more established at day 30 compared to day 15 in our organoid differentiation systems. These results enable us to use this computational phenotyping approach to compare wild type and genetically modified organoids.

### Generation of hiPSC lines and organoids carrying a genetic variant associated with Ebstein’s Anomaly

The homeobox-containing transcription factor *NKX2-5* plays a critical role in embryonic heart development^17,45^. Notably, *NKX2-5* knockout mice die at E10.5 with only two heart chambers, both with atrial identities as reported by ATLAS-seq predictions^22^. Furthermore, a single nucleotide variant in the *NKX*2-5 gene locus at the 673th nucleotide converting the 188th amino acid from Aspartate (N) to Lysine (K) was associated with Ebstein’s Anomaly, a congenital heart defect diagnosed with atrialized right ventricle and abnormal tricuspid valve^16,46^. We next used our two differentiation protocols for producing predominantly atrial and ventricular organoids (RA+ and RA- protocols), together with CRISPR/Cas9 technology, to characterize and study the effects of the above-mentioned genetic variant.

**Fig. 8:**
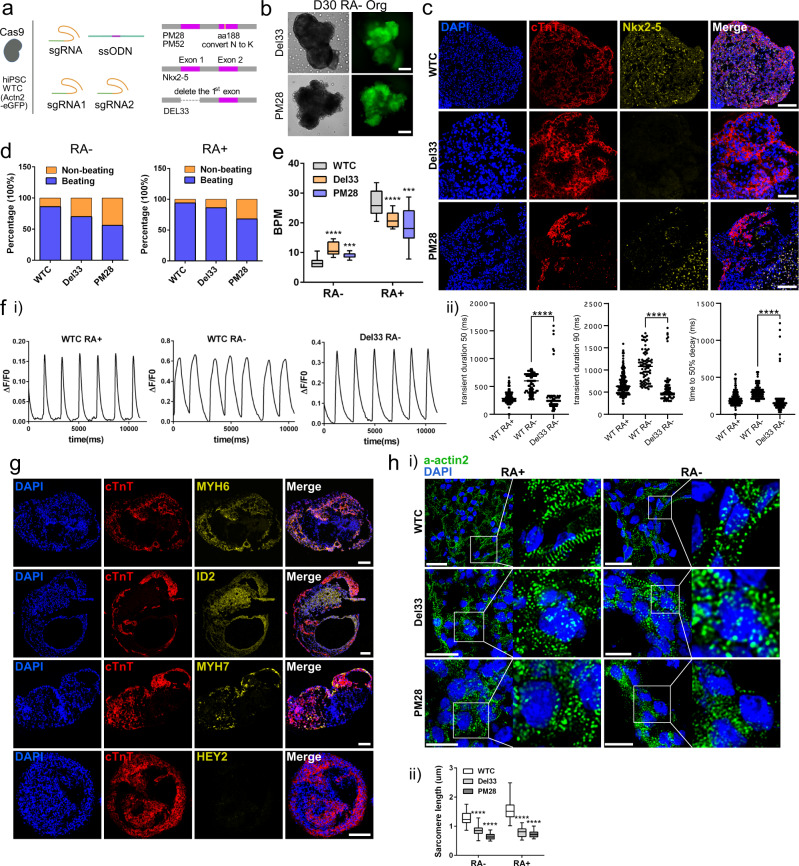
Nkx2-5 mutant heart organoids displayed functional and lineage defects. **a** Diagram of the strategy to generate an isogenic line carrying an EA-associated mutation (PM28, PM52) and a control line with a large deletion on Nkx2-5 (Del33). **b** Representative organoids differentiated from the two mutants at day 30. **c** Nkx2-5 expression significantly reduced in the Del33 line derived organoids. Scale bar = 100 μm. **d** The percentages of beating and non-beating organoids in the control and mutant at RA- and RA+ groups. *n* = 3 biological independent organoid differentiation. **e** The beating rates of control and mutant heart organoids. *n* = 20 organoids in controls, *n* = 20 and 10 organoids in Del33 RA+ and A-, *n* = 10 and 11 organoids in PM28 RA+ and A-, respectively. ***p < 0.001, ****p < 0.0001, Student’s *t* test against WTC. **f** (i) Representative plots of Ca^2 +^ transients in mutant and control organoid CMs at day 30. (ii) Quantification of the transient duration 50, transient duration 90 and time to 50% decay in WT and mutant organoid CMs. *n* = 81 cells in WT RA-, *n* = 208 cells in WT RA+, *n* = 77 cells in Del33 RA-. ****p < 0.0001, Student’s *t* test against WT RA-. **g** High expression of atrial genes and low expression of ventricular genes in RA- mutant CMs. Scale bar = 100 μm. **h** Defective sarcomere structures were identified in mutant heart organoids and sarcomere length was quantified based on the ACTN2-eGFP signal. *n* = 23 sarcomeres in each group, data was displayed as mean ± SEM. ****p < 0.0001, Student’s *t* test against WTC. Scale bar = 20 μm.

In order to do so, we produced an isogenic line introducing this mutation into the WTC line using a single-stranded oligodeoxynucleotide (ssODN) based CRISPR/Cas9 strategy and selected two clones (PM28 and PM52) for differentiation. As a control we created a line where the first exon of *NKX2-5* was deleted (Del33) using a pair of sgRNAs (Fig. 8a and Supplementary Table 3). We have confirmed the mutations and deletions using PCR and Sanger sequencing (Supplementary Fig. 12a, b). The reduction of Nkx2-5 expression in Del33 line-derived cells was confirmed by the flow cytometry analysis of Nkx2-5 expression on day 7 of differentiation (Supplementary Fig. 12c). After differentiation we observed ACTN2-GFP signal and beating cells in ventricular and atrial organoids at day 30 (Fig. 8b, Supplementary Fig. 1a, and Supplementary Video 3–6). Additionally, in the first exon deletion line we observed clear reduction of NKX2-5 immunostaining signal in the differentiated organoids (Fig. 8c).

We observed a lower percentage of beating organoids and ACTN2-GFP + cells in mutant lines compared to control lines under both RA+ and RA- conditions, indicating mutant lines have lower CM differentiation efficiency (Fig. 8d and Supplementary Fig. 12d). Next, we found the beating rates in the mutant organoids are higher than control (WTC line) organoids at RA- differentiation condition but lower at RA+ condition, while there is no significant differences in their organoid sizes (Fig. 8e and Supplementary Fig. 12e, f). Furthermore, we analyzed calcium transients and found the transient durations (TD50, TD90) in Del33 RA- organoids are close to the WTC RA+ organoids and clearly shorter than the WTC RA- organoids (Fig. 8f). Additionally, we analyzed mutant organoids with atrial and ventricular CM marker genes and found PM28 RA- organoids had high number of cells staining for atrial markers (MYH6, ID2)^29,36,37^ and fewer cells than expected staining for ventricular markers (MYH7, HEY2)^29,34,35^ (Fig. 8g). Similarly, we also found high number of ID2 staining positive cells and lack of HEY2 expression in Del33 RA- organoids (Supplementary Fig. 12g). Finally, we observed ectopic expression of smooth muscle gene MYH11 and defective sarcomere structures in the mutants (Fig. 8h and Supplementary Fig. 12h, i). All these analyses together suggested that *NKX2-5* plays multiple roles in heart development including the regulation of atrial-ventricular CM lineage specification, repression of non-cardiac lineage genes expression, and promotion of the sarcomere formation.

### Transcriptional analysis of mutant heart organoids

**Fig. 9:**
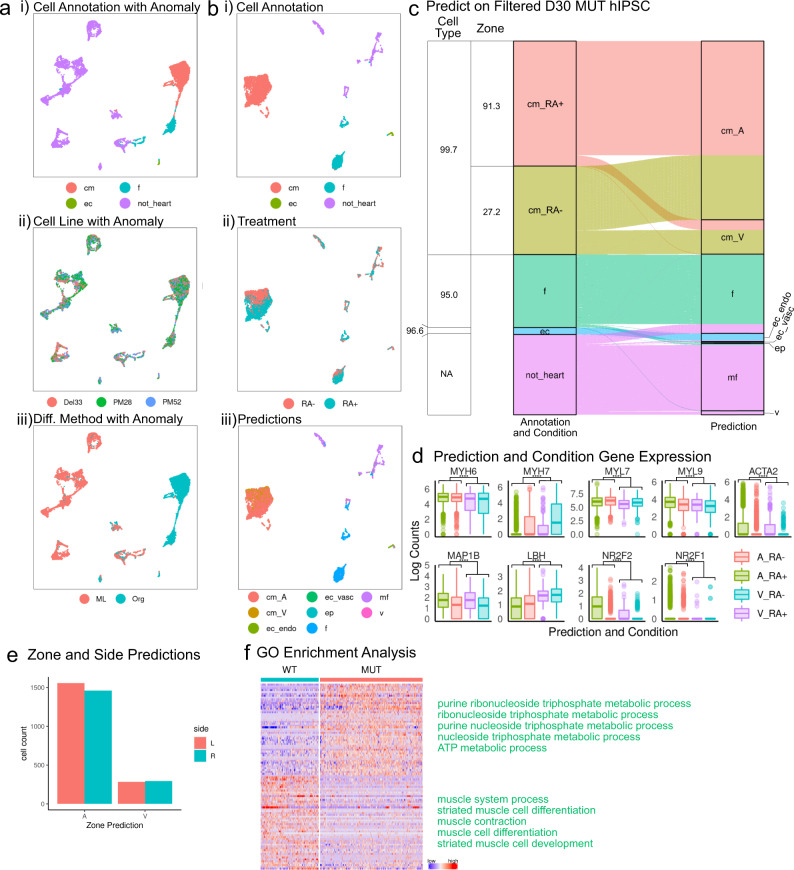
Study of EA defects in a mutant isogenic line using random forest predictions. **a** UMAP projections of mutant RA- and RA+ single cells at day 30 grouped by (i) cell types, (ii) cell line, and (iii) differentiation method. **b** UMAP projections of day 30 mutant RA- and RA+ single cells with Anomalies removed grouped by (i) cell types, (ii) treatment, and (iii) predicted cell types and zone. **c** Sankey diagram of prediction results of mutant RA- and RA+ cells at day 30 (Anomalies removed). Table provides cell type and conditional zone accuracies. **d** Expression levels of genes used to make prediction decisions that either correlate with the predictions. *N* = 1087 A_RA-, 407 V_RA-, 1931 A_RA+, 173 V_RA+. ****p ≤ 0.0001, Bonferroni corrected *p* values from Wilcox test, exact *p*-values and sample numbers available in a Source data file. **e** The results of side predictions in mutant cells at day 30. **f** Gene pathways that differentially enrich in the wild-type and mutant (predicted) CMs.

Within the same MULTI-seq setup as discussed above we profiled organoids from the PM28, PM52, and Del33 cell lines at day 30. After bioinformatics processing and quality control, we recovered 5596 (PM28), 3465 (PM52) and 4367 (Del33) cells for downstream analysis. Unsupervised clustering and (manual) marker gene analysis revealed major cardiac cell types (Fig. 9ai, bi and Supplementary Fig. 13). We did not observe pronounced differences between the three mutant lines (PM28, PM52, Del33, Fig. 9aii), but found cells from organoids were largely different from monolayer-differentiated cells (Fig. 9aiii), a signal that is driven by most monolayer-differentiated cells likely not being heart cells (Fig. 9ai, iii). Next, we used our classification approach to automatically annotate cell types and zones in this dataset. Consistent with previous results we found that anomaly detection predominantly filtered out non-heart cells, allowing us to exclusively focus on cardiac cell types (CMs, fibroblasts, and endothelial cells) for downstream analysis. Furthermore, predicted cell type annotations were highly consistent with our manually inferred cell types (average accuracy = 95%, Fig. 9biii, c). While, like wild-type cells, global transcriptional differences between RA- and RA+ differentiation protocols manifest mostly in CMs (Fig. 9bi, ii), the strong distinction between RA- and RA+ treated cells visually observed in wild-type CMs was lost (Figs. 7bii and 9bii). We also found a significant fraction of RA- (i.e., expected to be ventricular-lineage) CMs annotated as “atrial” by our label transfer procedure. While we did observe this type of “cross-classification” in the wild-type cell lines, the “cross-classified” fraction of cells in mutants significantly increased (72.8% here vs. 37.4% for wild-type cells from day 30 organoids, p < 2.2E-16, binomial test, Figs. 9c and 7c). Like before, we found atrial genes (including *MYL7*, *MYH6, MYL9*) highly expressed in predicted “atrial” CMs from organoids differentiated with the ventricular protocol (RA-) (Fig. 9d). Like in the wild-type cells, we observed laterality bias in CMs (Fig. 9e); however, for the modified lines we observed slightly more “right” ventricular-predicted cells, which was the opposite of wild-type cells (Fig. 7e).

Finally, when comparing gene expression between wild-type and mutant hiPSC-derived cells, we found that there were significantly more differentially expressed genes (DEGs, fdr<0.001) between wild-type and mutant cells in CMs (166), compared with fibroblasts (21), and endocardial endothelial cells (2) (Supplementary Data 5). In order to rule out the possibility that differences in cell type abundances account for this observation (we find 279 endocardial endothelial cells, 2158 fibroblasts, 5628 CMs), we down-sampled CMs to either 279 cells (like endocardial endothelial cells) or 2158 cells (like fibroblasts) 200 times and re-calculated DEGs. This procedure confirmed more DEGs in CMs compared to other cell types (Supplementary Fig. 14). This shows that the mutation of *NKX2-5* mainly affects CMs, and we see little evidence for effects on endocardial endothelial cells and fibroblasts. Focusing on CMs, Gene Ontology enrichment analysis of DEGs, highlights striated muscle development-related biological processes amongst the top down-regulated genes (low expression in mutant CMs), while the most significantly enriched terms in up-regulated genes (high expression in mutant CMS) highlight processes related to metabolism and energy (Fig. 9f and Supplementary Fig. 15).

Overall, these results demonstrate that we successfully generated ventricular-lineage and atrial-lineage organoid cells with a specific genetic variant associated with Ebstein’s Anomaly. Our classifier predicted most mutant CMs differentiated using the RA- protocol as atrial CMs, recapitulating the atrialized ventricular defects in Ebstein’s anomaly. Furthermore, our observations suggested that the *NKX2-*5 mutation predominantly impacts cardiomyocytes, with genes related to striated muscle differentiation showing weaker expression in mutant CMs, while genes related to metabolism and energy production appear up-regulated. This for the first time provides clear evidence that the Ebstein’s anomaly-associated variant (c.673 C > A)^16^ affects the expression of energy-related and key heart muscle genes during (in vitro) cardiogenesis, building confidence in this particular variant’s relevance and laying the foundation for more detailed disease models of Ebstein’s anomaly.

## Discussion

In this study, we generated ventricular-lineage and atrial-lineage heart organoids and used scRNA-seq in combination with MULTI-seq sample pooling to obtain transcriptional profiles at single-cell resolution^28,39^. We established a machine learning label transfer method that allowed us to leverage annotations (cell type, anatomical compartment, laterality) from primary human fetal cells, and we used this approach to characterize cells differentiated with our organoid systems. Finally, we used this experimental and computational combination to compare differentiated organoids from wild-type cell lines with organoids carrying a genetic variant associated with Ebstein’s anomaly, effectively establishing variant-specific in vitro hiPSC model for this type of congenital heart defect.

We find that our organoid systems recapitulated the microenvironment of human developing hearts by self-assembling into chamber-like structures. We note that this type of three-dimensional approach has advantages when studying heart developmental processes, especially chamber formation, compared to monolayer and co-differentiated microtissue systems^47,48^. We note that along the same lines Hofbauer et al.^3,49^ recently reported that the addition of VEGF to cardioids (a similar type of cardiac organoid) can lead to the development of endothelial cells that comprise the entire inner layer of chambers, essentially equivalent to the in vivo anatomical pattern observed in endocardial endothelial cells^49^. With this study and our work generating specific cell types in environments approximating their in vivo anatomical compartments, it will be interesting to establish differentiation protocols in the future that mimic the in vivo localization of other cardiac cell types, like epicardial cells and vascular endothelial cells.

Based on the sarcomere lengths and scRNA-seq data, we found that hiPSC-derived CMs in both organoid and monolayer systems were relatively immature, compared to primary cells. Additionally, we found CMs in organoids were slightly less mature compared to CMs in monolayer system (Supplementary Fig. 11 and Supplementary Data 4). Currently we do not know if this is specific to our protocol, or if organoids from other hiPSC protocols^11–13^ share this characteristic; comparison of these organoids to appropriate monolayer CMs could elucidate this issue and contribute to our understanding of cell maturation in 2D vs. 3D differentiation protocols more generally. Considering it has been reported that co-culture of CMs and other cardiac cells (fibroblasts, endothelial cells) can improve CM maturation, we believe that organoid cells may further mature after long-term culture^50^. Furthermore, cells often become more mature after extensive culture, as has been reported for other types of organoids such as brain^51^. We note, though, that this phenomenon is likely tissue- or organ-specific, because kidney organoids did not mature after further culturing, but instead showed higher cell death rate^52^. However, it is known that in vitro differentiated CMs can mature after being transplanted into live organisms^53^. While it would be challenging to transplant generated heart organoids to replace the heart of model organisms, it may be feasible to transplant them into other parts of animal models, like mice or zebrafish, to study their maturation.

The organoids we generated for this study were differentiated directly from hiPSCs, which is similar to the in vivo fetal heart developmental process. In addition, we demonstrated the potential to approximate critical heart structures like chambers, rendering this method a strong candidate for modeling normal heart development and congenital heart defects, a key difference compared with non-hiPSC-based organoid methods^54,55^. Additionally, we note that, compared to the other hiPSC-based organoid methods, our method is based on microwells and can generate the organoids in a very high throughput way. Importantly, we have optimized our protocols to generate atrial-lineage and ventricular-lineage organoids, which has not been reported in other methods. This makes our approach uniquely suited to study atrial and ventricular lineage developmental heart defects.

Through transverse section analysis of organoids, we found they are heterogeneous and can be grouped into three categories: intact, holes, and cavities. Further quantification of the prevalence of these categories revealed their percentages are similar across multiple differentiation batches. Categorizing our organoids highlights the ability of some organoids to recapitulate heart structures like chambers (cavities) while others do not (holes and intact). Additionally, categorizing will help us tune our protocols for category-specific creation of organoids in the future. Our preliminary data along with other recent publications have shown that different Wnt and BMP signaling concentrations at cardiac mesoderm stages can change the proportion of organoid categories^3^. Specifically, in our preliminary data we found the relatively higher CHIR concentration can likely promote the development of organoids with cavity structures (Supplementary Fig. 16).

Immunofluorescence analysis of cardiac cell type marker genes revealed two fibroblast distributions in organoids. In a majority of organoids, fibroblasts were seen to mix with cardiomyocytes forming a similar distribution as observed in vivo, while in a small proportion of organoids, fibroblasts clustered separately from cardiomyocytes. Additionally, we note that our scRNA-seq data indicates fibroblasts in our organoids are transcriptionally similar to cardiac fibroblasts; this suggests they may be able to perform some functions of cardiac fibroblasts, like secreting growth factors and extracellular proteins to regulate CM development and maturation. Therefore, like our results for Ebstein’s anomaly show, our approach is suited to model structure-related CHDs.

While in silico phenotyping of organoid cells performed well overall, the experimental setup with different platforms for training (STRT-seq) and testing/application (10X) is clearly not optimal, and generating more similar test/training data in future experiments will likely increase accuracy and reliability of computational phenotyping. In addition, making use of spatial transcriptomics approaches to increase resolution and confidence of annotations with regard to anatomical zone and laterality, without the need for tissue dissections, would yield an increased chance of capturing more subtle transcriptional spatial features. When predicting anatomical zones for wild-type organoid cells, the vast majority of predictions agreed with the differentiation protocol (atrial = RA+ , ventricular = RA-, see Fig. 7c). However, for a smaller group of cells the protocols and predictions mismatch, that is RA- differentiated cells were predicted “atrial” and RA+ differentiated cells were “ventricular”. Further investigating those mismatching cells yielded zone-specific genes that support zone predictions (e.g., atrium-specific genes *MYL7* and *MYH6* are high in RA- differentiated but “atrial” predicted cells) and others more in-line with the differentiation protocols (e.g., ventricle-specific genes *PLN*, *MEIS2*). In the future it will be interesting to further investigate these genes, and more specifically elucidate their relation to RA, the only difference between the atrial and ventricular differentiation protocols. However, to assess whether direct regulation by RA plays a role will require further experiments, like applying ChIP-seq or derivative technologies in generated organoids^56^.

We also noted that the fraction of heart cells we recovered by scRNA-seq varied between the monolayer (low fraction of heart cells recovered) and organoid protocols (high fraction of heart cells recovered), see Figs. 2f, 4e. Exception to that rule were the WTC organoids at day 15. Since monolayer protocols have been reported to generate cardiomyocytes with high efficiency, we believe this observation may be specific to this batch rather than being representative of the monolayer approach. In addition, we have compared cells from low-efficiency monolayer differentiation with monolayer cells from other labs where scRNA-seq data has been made available and found that label transfer works well (Supplementary Tables 1 and 2. These results suggest CMs from our monolayer differentiations display typical transcriptional hallmarks. Therefore, we interpret the data in the sense that it shows our organoid protocol to be efficient.

Our protocols also allowed us to produce genetically modified cell lines carrying a mutation associated with Ebstein’s anomaly, and to compare resulting organoids with wild-type differentiations. We found that genes down-regulated in mutant organoids were associated with striated muscle differentiation, while mutant up-regulated genes were often related to energy and metabolism, which provided the first (in vitro) characterization of molecular effects of the *NKX2-5* c.673 C > A mutation and may constitute a first step towards more detailed models of the contribution of this genetic lesion towards Ebstein’s anomaly. We note, however, that in addition to atrial/ventricular CM lineage defects, Ebstein’s anomaly patients also present with tricuspid valve defects. Our current system does not model this aspect, but we can extend our approach (differentiation system and cell type/anatomical zone computational modeling) to include and focus on valve-related cells in the future. Furthermore, Ebstein’s anomaly is known to be genetically multigenic^14,57^, and our general approach of in vitro modeling together with computational phenotyping can be applied to other Ebstein’s anomaly-associated genetic variants to gain systematic insight into the disease.

In summary, in this work we have established chamber-specific differentiation protocols for heart organoids, and we showed that in combination with scRNA-seq profiling of organoid cells this system is a useful model for investigating genetic lesions at the *NKX2-5* locus associated with Ebstein’s anomaly. While it was necessary to focus on zone/chamber specificity (atrial vs. ventricular) in this context, our approach can be repurposed to focus on laterality (left vs. right), which would be interesting in the context of CHDs with known laterality phenotypes, such as heterotaxy and hypoplastic left or right heart syndrome.

## Methods

### Maintenance of hiPSC lines

WTC line with ACTN2-eGFP transgene (Coriell catalog: AICS-0075-085), WTC line with MYL2-eGFP transgene (Coriell catalog: AICS-0060-027), WTC-mTagRFPT-CAAX-Safe harbor locus (AAVS1)-cl91 (Coriell catalog: AICS-0054-091) and SCVI114 line (Gift from Stanford CVI) were maintained in completely defined albumin-free E8 medium (DMEM/F12 with l-glutamine and HEPES, 64 μg/ml l-Ascorbic Acid-2-phosphate, 20 μg/ml insulin, 5 μg/ml transferrin, 14 ng/ml sodium selenite, 100 ng/ml FGF2, 2 ng/ml TGFb1)^58^ on Matrigel (Corning, CB40230A) coated tissue culture plates. Medium was changed daily and routinely passaged every three to four days using 0.5 mM EDTA solution (Invitrogen, 15575020). 10 μM ROCK inhibitor Y27632 (Selleckchem, S10492MG) was supplemented to the medium during cell passaging.

### Monolayer cardiac differentiation

Monolayer cardiac differentiation was carried out following a published protocol^59^. Briefly, RPMI 1640 media (Corning, 10040CVR) was used as the basal medium in the entire differentiation process. B-27 Supplement minus Insulin (Gibco, A1895601) was supplemented to the medium for the first 6 days and B-27 Supplement (Gibco, 17504044) was used afterwards. The small molecule inhibitor of GSK3β signaling, CHIR99021 (Selleckchem, S292425MG) was used in the first two days of differentiation and Wnt signaling inhibitor C59 (Selleckchem, S70375MG) was added from day 3 to day 4. To differentiate atrial cells, 1 uM retinoic acid (Sigma-Aldrich, R2625) was added from day 3 to day 6 as described Previously^42^.

### Cardiac organoid differentiation

The cardiac organoid differentiation procedure was adapted from a protocol described Previously^2^. Briefly, 1.5×10^6^ hiPSCs were seeded in each well of AggreWell™800 plates (STEMCELL, 34815) according to the manufacturer’s instructions. The cells were assembled into 3D structure by culturing in E8 medium for 2 days (day -2 to day 0). From day 0 to day 6, cells were cultured in RPMI supplemented with B-27 minus insulin. CHIR99021 at a final concentration of 11 µM was used at day 0 and lasted for 1 day. To analyze the function of WNT signaling in regulating the development of organoid internal structures, different CHIR concentrations (4, 8, 11, 15 μM) were applied at this step and the differentiations were carried out with WTC hiPSC line (AICS-0054-091). From day 3 to day 5, cells were treated with C59 at a final concentration of 5 µM. The cell aggregates were transferred to 5% Poly(2-hydroxyethyl methacrylate) (Sigma-Aldrich, P3932) coated tissue culture plates at day 7 and cultured in RPMI with B27 supplement until the end of differentiation. Fresh medium was changed every 3 days until tissue harvest. To differentiate organoids, 1 uM retinoic acid was added from day 4 to day 7.

### Generating Nkx2-5 mutant hiPSC lines

To generate Nkx2-5 loss of function mutants, a pair of single-guide RNAs (sgRNAs) (Supplementary Table 3) were used to target the first exon of Nkx2-5 gene. The sgRNAs were cloned into pSpCas9(BB)-2A–GFP (PX458) vector and transfected into WTC hiPSCs with nucleofector. Specifically, about 8 × 10^5^ iPSCs were transfected with 5 μg of plasmids with Lonza Human Stem Cell Nucleofector Kit 1 (Lonza, VPH-5012) on a Nucleofector 2b device (Lonza, AAB1001). After FACS sorting and PCR genotyping of multiple iPSC clones, we identified the clones with the deletion of first Nkx2-5 exon and further expanded them for cardiac differentiations.

Besides, we introduced a single nucleotide mutation into the Nkx2-5 gene locus using a ssODN-based CRISPR strategy. We co-transfected a ssODN and sgRNA (Supplementary Table 3) to convert the 673^th^ nucleotide from C to A, which led the protein change at 188 amino acid from Asn to Lysin. After clone picking and genotyping the clones by sanger sequencing, we identified the positive clones and further expanded them for differentiation.

### Single-cell isolation

Cardiac cells from monolayer differentiation culture at day 15 and day 30 were washed twice with PBS and incubated with TrypLE Express (Life Technologies, A1217702) for 15 min at 37 °C. Cells were collected by centrifuge at 300 g for 5 min and washed once with HBSS-/- (Ca^2+^/Mg^2+^ free). The cells were further resuspended in 1 ml B27 and filtered through a 40 μm filter (Corning, 431750). After that, the cells will be ready for FACS sorting or directed used for scRNA-seq.

The cardiac organoids were collected and washed twice with HBSS−/- (Ca^2+^/Mg^2+^ free) before being incubated with 0.25% Trypsin/EDTA at 37 °C for 5 min. After that, a collagenase HBSS+/+mixture with 10 mg/ml of collagenase A (Sigma-Aldrich, 10103586001), 10 mg/ml of collagenase B (Sigma-Aldrich, 11088815001), and 40% FBS (Gibco, 26140079) was added to the digestion solution and gently pipetted until the organoids were completely dissociated. The cells were then spun down at 300 × g for 5 min, resuspended in 1 ml of RPMI/B27 medium, and filtered through a 40 μm filter.

### MULTI-seq barcoding

First batch (FACS sorted). The cells from monolayer differentiations and organoids were washed once with FACS buffer (HBSS-/-,10% FBS) and resuspended in 1 mL of FACS buffer with 10 μM ROCK inhibitor. After FACS sorting based on GFP expression (BD, FACSAria™ III), both GFP positive and negative cells were collected separately for scRNA-seq. Each sample was stained with a unique MULTI-seq barcode following a published protocol with minor modifications^28^. Briefly, the cells were washed twice with PBS (Ca^2+^/Mg^2+^ free) and resuspended in 180 μl PBS (Ca^2+^/Mg^2+^ free). 20ul of 2 μM sample-specific Anchor/Barcode (Supplementary Table 4) was then added to each sample and incubated for 5 min on ice. After that, 20 μl of 2 μM Co-Anchor solution (Supplementary Table 4) was added and kept on ice for another 8 mins. The samples were then washed once and resuspended in ice-cold PBS with 1% BSA. The cell numbers were counted before pooling the samples together.

Second batch (Not sorted). All cells including the monolayer differentiated cells and organoid differentiated cells (WTC and SCVI111 line differentiated in atrial and ventricular differentiation protocols at differentiation day 15 and 30, and Nkx2-5 mutant line in atria and ventricular differentiation protocols at day 30) were prepared as single cells. The cells in each sample were stained with MULTI-seq barcode following the same procedure as the first batch of cells. Afterwards, the cells were counted and pooled together for scRNA-seq.

### Library preparation and single-cell mRNA sequencing

The pooled cells were captured in 10X Chromium (10X Genomics, 120223) by following the single-cell 3’ reagent kits v3 user guide. Briefly, cells were loaded into each chip well to be partitioned into gel beads in emulsion (GEMs) in the Chromium controller. We targeted for 25,000 cells in each chip well and profiled one well for the first batch experiment and two chip wells for the second experiment. The cells were then lysed and barcoded reverse transcribed in the GEMs. After breaking the GEMs and further cleanup and amplification, the cDNA was enzymatically fragmented and 3’ end fragments were selected for library preparation. After further processing including end repair, A-tailing, adapter ligation, and PCR amplification, a string of sequences including sample index, UMI sequences, barcode sequences, and sequencing primer P5 and P7 were added to cDNA on both ends. The libraries were sequenced on Illumina HiSeq × platform.

### Bioinformatics analysis

#### Data processing and quality control

Alignment and quantification of UMI counts for endogenous genes were performed using the cellranger count pipeline of the Cell Ranger software (version 3.1.0). We used the human reference genome (GRCh38.p12) and arguments --chemistry = SC3Pv3 and --expect-cells as 10,000 or 25,000, depending on the specific library. For sample demultiplexing, we used the R package deMULTIplex (version 1.0.2, https://github.com/chris-mcginnis-ucsf/MULTI-seq) which consists of alignment of the MULTI-seq sample barcode read sequences to the reference MULTI-seq sample barcodes followed by sample classification into doublets and singlets. Multiple quality control (QC) metrics were calculated using the R package scater (http://www.bioconductor.org/packages/release/bioc/ html/scater.html), and cells with total library size > = 2000, number of detected genes > = 1000 and < = 8000, and < = 30% percentage of mitochondrial reads were considered. To account for doublets with the same MULTI-seq barcode we used the scds R package (https://github.com/kostkalab/scds) as described below. We focused on genes with one or more count in at least five cells (assessed for each batch separately) and calculated log-normalized counts using the deconvolution method of the scran R package (https://bioconductor.org/packages/release/bioc/html/scran.html). There are two batches in the unsorted data, so multiBatchNorm from the package batchler (https://bioconductor.org/packages/devel/bioc/html/batchelor.html) was used to perform scaling normalization so that the size factors are comparable across batches. Next, clustering, dimensionality reduction, and cell type annotation was performed separately on wildtype atrial, wildtype ventricular, and mutant groups. The top 2000 highly variable genes were identified using the modelGeneVar function (scran R package). Using these genes, 50 principal components were calculated (runPCA, scater R package) and used to generate UMAP plots (runUMAP, scater R package) and to build a shared nearest neighbor graph followed by walktrap clustering (cluster_walktrap, igraph R package, https://github.com/ igraph/igraph) as outlined by Amezquita et al^60^.

For FACs sorted data no batch correction was needed, so clustering was performed by building a shared nearest-neighbor graph using the first 25 first principal components for each cell; we used Jaccard weights and the Louvain clustering algorithm from the igraph package with steps = 10 parameter. The R package ComplexHeatmap (http://www.bioconductor.org/packages/release/bioc/html/ComplexHeatmap.html) was used to generate gene expression heatmaps and findMarkers (scran R package) with the fdr = .001 parameter was used to get inter-cluster differentially expressed genes. Finally, cell type annotations were manually resolved using cluster expression patterns of the following genes: *TNNT2, ACTN2, TNNI3, TTN, MYH6, NR2F2, MYL2, MYH7, COL1A1, DCN, SOX9, POSTN, WT1, TBX18, ALDH1A2, LRRN4, CSF1R, TPSAB1, CD3D, GIMAP4, PECAM1, CDH5, TIE1, NPR3, PLVAP, FOXC1, FABP4, CLDN5, HEMGN, HBA-A1, HBA-A2, C1QA*. In the rare case where a cluster expresses marker genes for more that one cell type, iterative clustering was performed to resolve cell types.

#### Computational annotation of multiplets in a MULTI-seq workflow

The MULTI-seq approach identifies multiplets based on occurrence of more than one MULTI-seq cell barcode. By design, this approach cannot identify mutiplets comprised of cells with identical MULTI-seq sample barcodes. We use computational multiplet identification (scds) to identify this type of “within-sample” multiplet computationally. Broadly, we use MULTI-seq data to estimate the fractions of within-sample and between-sample multiplets and use them to determine the number of within-sample multiplets that we annotate computationally.

Specifically, in our approach we assume the overall fraction of cells (We use “cell” as a shorthand for cell/10X-barcode in an abuse of notation, since multiplets are not single cells by definition) being multiplets, ${p}_{m}$, is comprised of within-sample multiplets (${p}_{w}$, with the same MULTI-seq barcode) and between-sample multiplets (${p}_{b}$, with distinct barcodes) and no other contributions: ${p}_{m}={p}_{w}+{p}_{b}={p}_{m}\left({p}_{w}/{p}_{m}+{p}_{b}/{p}_{m}\right)={p}_{m}{{{{{{\rm{\pi }}}}}}}_{w}+{p}_{m}{{{{{{\rm{\pi }}}}}}}_{b}$, where ${{{{{{\rm{\pi }}}}}}}_{w}$ and ${{{{{{\rm{\pi }}}}}}}_{b}$denote the fraction of multiplets being within-sample and between-sample, respectively; also: ${{{{{{\rm{\pi }}}}}}}_{w}+{{{{{{\rm{\pi }}}}}}}_{b}=1$. We then use the following ansatz, where the fraction of different types of multiplets is proportional to the abundance of constituent cells (we only focus on doublets and assume higher-order multiplets to be rare):${{{{{{\rm{\pi }}}}}}}_{w}\propto {\sum }_{j}{N}_{j}^{2}/{N}^{2}$ and ${{{{{{\rm{\pi }}}}}}}_{b}\propto {\sum }_{(i,j),i > j}{N}_{i}{N}_{j}/{N}^{2}$ where $N$ denotes the overall number of cells and ${N}_{k}$ the number of cells with MULTI-seq/sample barcode $k$. Utilizing estimates of these quantities obtained by demultiplexing MULTI-seq data and the constraint that ${{{{{{\rm{\pi }}}}}}}_{w}$ and ${{{{{{\rm{\pi }}}}}}}_{b}$ sum to one we obtain estimates ${\hat{{{{{{\rm{\pi }}}}}}}}_{w}$ and ${\hat{{{{{{\rm{\pi }}}}}}}}_{b}$.

Let ${D}_{m}$ be the number of MULTI-seq annotated between-sample multiplets. We have ${D}_{m}=N{p}_{m}{{{{{{\rm{\pi }}}}}}}_{b}$ and therefore ${p}_{m}={D}_{b}/\left(N{{{{{{\rm{\pi }}}}}}}_{b}\right)$ and plugging in ${\hat{{{{{{\rm{\pi }}}}}}}}_{b}$ yields ${\hat{p}}_{m}$, an estimate for the fraction of doublets in our data set; note that ${\hat{p}}_{m} > {D}_{b}/N$, the fraction of multiplets obtained from the MULTI-seq data alone. The “missing” number of within-sample multiplets is then estimated as $N{\hat{p}}_{m}{\hat{{{{{{\rm{\pi }}}}}}}}_{w}$, determining the number of doublets we annotate using scds in addition to the between-sample doublets annotated by MULTI-seq.

#### Random forest based cell type classification across data sets

##### Training data

We used the single-cell RNA-seq data of Cui et al.^29^ with cell type, anatomical zone, and laterality annotations in order to train a random forest classifier that generalizes to other data sets. Data and cell annotations were downloaded from GEO (GSE106118)^29^. Iterative clustering was used to further resolve the annotated endothelial cells into endocardial endothelial cells and vascular endothelial cells based on the expression of endocardial endothelial cell markers (*NPR3*, *PLVAP*, *FOXC1*) and vascular endothelial cell markers (*FABP4* and *CLDN5*). Additionally, iterative clustering was used to re-annotate a subcluster of “5w” cells as epicardial cells based on the expression of *WT1*, *TBX18*, *ALDH1A2*, *LRRN4*, and *UPK3B*. Because there were so few (only 27) mast cells annotated in this dataset, they were not used to train the model. Lastly, only cells from the left ventricle, right ventricle, left atria, and right atria were used. The final cell type annotations used are provided in Supplementary Data 6. These cells form the input for downstream analyses and classifier training.

##### Development data set 1

We used the single-cell RNA-seq data of Asp et al. with zone and cell type annotations to validate our random forest model^30^. Data and cell annotations were downloaded from https://www.spatialresearch.org/resources-published-datasets/doi-10-1016-j-cell-2019-11-025/. Cells annotated as cardiac neural crest were re-annotated as immune cells based on high expression of *C1QA*, *CSF1R*, and *GIMAP4*. The final cell type annotations used are provided in Supplementary Data 7 In order to compute log-transformed normalized expression values, clusters were first computed (quickCluster, scran R package), followed by normalization where size factors are deconvoluted from clusters (computeSumFactors, scran), followed by log-transform normalization (logNormCounts, scater R package).

##### Development data set 2

We used the single-cell RNA-seq data of Miao et al. with laterality annotations to validate our random forest model^23^. Data were downloaded from GEO (GSM4125587, GSM4125585, GSM4125586, GSM4125588). In order to compute log-transformed normalized expression values were computed as for development data set 1. Furthermore, highly variable genes, low dimensional embeddings, clustering, and cell type annotations were performed as for the unsorted hiPSC data set (see Data processing and quality control). The final cell type annotations used are provided in Supplementary Data 8.

##### Model fitting

To fit a model on the training set and apply it to a test data set, typically generated with different platform technology, we proceed as follows. First highly variable genes in both data sets were selected. Using the modelGeneCV2 function (scran R package) we fit the squared coefficient of variation (CV^2^) and the top 50% of genes with the largest CV^2^ and strongest deviation from the fit line were retained as highly variable genes. Additionally, genes expressed in less than 1/4th of cells were filtered out. Gene passing both filters on the train and test data were scaled (for each data set independently) and kept for RF model fitting. Genes were scaled by subtracting their minimum expression value, and then dividing by their 95th quartile. Next, we used the R package ranger (https://cran.r-project.org/web/packages/ranger/index.html) to derive a random forest classifier on the scaled train data (impurity importance score), using class weights to account for imbalances between cell-type labels. We then use the top genes in terms of feature importance to train a second, final random forest on the train data, which is then used to derive labels on the scaled test data set. Hyper parameters for this procedure (number of trees, number of genes for the second round of learning) were determined separately using the training data as both, test and train set, respectively. To optimize a parameter, the others were held constant while a range of values was tested and the final value was selected as the elbow point when plotting accuracy against tested parameter values. Next, the trained model is used to predict the labels on a test set. Performance is visualized using Sankey diagrams (ggplot2, https://github.com/cran/ggplot2). Cell type accuracies are calculated as the percentage of correctly classified cells. Conditional accuracies were calculated as the percentage of correctly classified cells within a given label.

##### Cell type classification

For cell type classification all cells in the training data were used in the above procedure with 300 trees and 500 important genes (Supplementary Data 9) as hyperparameters.

##### Anatomical zone classification

Here we focus on the anatomical zone of cardiomyocytes (CMs), and correspondingly only CMs are used in the above procedures. Hyper parameters used are: 300 trees and 100 important (Supplementary Data 9) genes for the second random forest.

##### Laterality classification

Here we focus on the laterality (left/right) of CMs; we proceed as discussed above, with an additional quantile normalization step after determining top-variable genes and before scaling. Hyper parameters we determined were 500 trees and 100 important genes (Supplementary Data 9).

##### Anomaly detection

To flag cells in the hiPSC data the model has not seen before we perform anomaly detection as follows: Cell type classification (see above) was performed and for each cell the annotated class and its class-probability were recorded. If that probability was lower than a class-dependent threshold the cell was considered an anomaly. The threshold for each class was determined as the minimum of the two 5% quantiles of probabilities of cells in the corresponding class in the two development sets^23,30^.

#### SingleR label transfer

To investigate whether our monolayer CMs are transcriptionally similar to monolayer CMs generated in other studies with higher differentiation efficiencies, we used the R package SingleR to transfer cell type annotations from the other studies to our data^61^. First, we obtained two datasets from previous monolayer studies by Churko et al. and Friedman et al^40,41^. Day 14 and day 45 single-cell data from Churko et al. were acquired using synapse ID: syn7818379 while day 15 and day 30 single cell data from Friedman et al. were acquired from the filtered 10X matrices downloaded from EMBL-EBI under accession number E-MTAB-6268. Both datasets were preprocessed as follows. First, genes that were not expressed in at least one percent of the cells were filtered out. Then, in order to compute log-transformed normalized expression values, clusters were first computed (quickCluster, scran R package), followed by normalization where size factors are deconvoluted from clusters (computeSumFactors, scran), followed by log-transform normalization (logNormCounts, scater R package). Next, clustering was performed as for the unsorted hiPSC data set (see Data processing and quality control). Lastly, each cluster was assigned cell types “cm” and “other” based on the expression of CM marker genes TNNT2, ACTN2, TNNI3, and TTN (clusters that highly expressed these marker genes were labeled as “cm” and everything else was labeled “other”). We then used the “SingleR” package to transfer these labels onto our monolayer dataset. Labels from these previously generated monolayer datasets correctly transfer onto our data, which indicates our CMs are transcriptionally similar despite low differentiation efficiency (Supplementary table 2 and 3**)**.

#### Single molecular in situ hybridization

To visualize the transcriptional expression patterns of Tnni3, Cdh5, Postn, and Wt1 in the organoids, proximity ligation in situ hybridization (PLISH) was performed as previously described with minor modifications^62^. Briefly, the organoids were fixed with DEPC treated 4% paraformaldehyde (electron microscopy sciences, 15710 S) before being embedded with OCT (Sakura, 4583). The embeded tissue were then sectioned with the thickness of 6 μm and treated with post-fix medium (3.7% formaldehyde (Sigma-Aldrich, 252549) and 0.1% DEPC (Sigma-Aldrich, D5758) for 30 min. After that, the sections were incubated with hybridization buffer (1 M NaTCA, 5 mM EDTA, 50 mM Tris pH 7.4, 0.2 mg/mL Heparin) and H probes (Supplementary Table 5). After circulation ligation and rolling circle amplifications, the detection probes conjugated with Cy3 or Cy5 fluorophore were applied and the hybridization signal were imaged under confocal microscopy (Leica TSC SP8).

#### Immunofluorescence staining

Organoids were fixed in 4% paraformaldehyde (electron microscopy sciences, 15710 S) for 1 hr. After that, the organoids were washed twice with PBS and embedded in OCT. The tissues were sectioned at 6 μm and used for staining. The immunostaining procedure was carried out as previously described^63^. Briefly, the section slides were washed with PBS for 5 min and permeabilized with PBST (0.2% Triton X-100 in PBS) for 10 min. After that, the slides were sequentially incubated with blocking buffer (10% Goat Serum, 1% BSA, 0.1% Tween 20) for 1 h at room temperature and primary antibody in PBST with 1% BSA overnight at 4°C. The antibodies were diluted according to the manufacturer’s instructions. The mouse anti-Cardiac Troponin T (5 µg/ml, Invitrogen, MA5-12960), mouse anti-human COUP-TF II/NR2F2 antibody (1:1000, R&D Systems, PP-H7147-00), rabbit anti-MYL7 antibody (1:1000, Sigma, SAB2701294), rabbit anti-Cardiac Troponin T (1:400, Abcom, ab45932), mouse anti- MYH6 (1:50, DSHB, S46), mouse anti- MYH7 (1:50, DSHB, BA-D5), mouse anti- ID2 (1:50, DSHB, PCRP-ID2-1A8), mouse anti- HEY2 (1:50, DSHB, PCRP-HEY2-1H10), mouse anti- COL1A1 (1:50, DSHB, SP1.D8), mouse anti-NFATC1 (1:25, DSHB, PCRP-NFATC1-1A2), rabbit anti-VE-Cadherin (1:400, Cell signaling, #2500), mouse anti-Nkx2-5 (25 μg/ml, R&D Systems, # MAB2444), rabbit anti-Myosin, Smooth Muscle Heavy Chain (1;200, Biomedical Technologies, BT-562), mouse anti-MF20 (1:100, DSHB, MF 20) were used. The slides were further washed three times with PBS and incubated with secondary antibodies in blocking solution for 1 h at room temperature. The secondary antibodies used include goat anti-mouse 488 (5 μg/ml, A11001, Invitrogen), goat anti-mouse 594 (10 μg/ml, A11005, Invitrogen), Goat anti-Rabbit 568 (5 μg/ml, A11036, Invitrogen), Goat anti-Mouse 647 (5 μg/ml, A21235, Invitrogen) and goat anti-rabbit 647 (10 μg/ml, A-21245, Invitrogen). Finally, the slides were stained with DAPI (Thermo Scientific, 62248) for 5 min and mounted using ProLong^TM^ Diamond Antifade Mountant (Molecular Probe, P36962). The Images were captured using Leica TCS-SP8 confocal microscope. For the quantification of the cardiac Troponin T (cTNT) expression, the mean gray values of cTnTsignal were measured using Fiji^64^ and normalized to the whole area of organoid.

#### Organoid imaging and processing

The images of beating organoids were taken under Leica DMI6000 microscope, three to ten of images were used to measure the organoid diameters. The length of both longest axis and shortest axis were measured for each organoid. We grouped the organoids based on the diameters of their internal empty structures which were analyzed under microscope with their section images. For the organoids with a diameter less than 50 μm, we defined them as “intact”; For those with size between 50 to 100 μm, we called them as “holes”; For those with diameters above 100 μm, we identified them as “cavities”. Besides, the beating organoids were recorded at an interval of 50 ms with a Hamamatsu Orca-ER camera with transmitted light. The beating rates were calculated with beats/frames multiplied by frames/second.

#### Flow cytometry

The organoids were dissociated through sequential incubation with 0.25% Trypsin/EDTA and a 10 mg/ml collagenase HBSS+/+ mixture. For the WTC line with ACTN2-eGFP reporter, the cell suspensions were directly used to quantify the ACTN2-eGFP cell percentages using BD LSR Fortessa analyzer. For Col1a1 staining, cells were fixed for 15 min at 4 °C with 4% PFA in PBS followed by permeabilization using ice-cold 90% methanol for 10 min. Cells were washed twice with PBS and stained with primary antibody (mouse anti-Col1a1, 1:50, DSHB, SP1.D8) in antibody dilution buffer (0.5% BSA in PBS) for 1 h at 4 °C. For Nkx2-5 staining, cells were fixed and permeabilized as described above, then stained with mouse anti-Nkx2-5 (1:100, R&D Systems, MAB2444) for 40 min at room temperature. For VE-Cadherin staining, cells were stained with primary antibody (rabbit anti-VE-Cadherin, 1:400, Cell signaling, #2500) for 1 h at 4 °C in antibody dilution buffer.

After staining with primary antibodies, the cells were washed with PBS and stained with fluorochrome-conjugated secondary antibody (diluted in antibody dilution buffer) for 30 min at 4 °C. The following secondary antibodies were used: goat anti-rabbit 647 (10 μg/ml, A-21245, Invitrogen), and Goat anti-Mouse 647 (5 μg/ml, A21235, Invitrogen). After washing twice with PBS, the stained cells were analyzed using the LSR Fortessa Analyzer (BD) or FACSAria™ III Cell Sorters (BD). Data were analyzed using FACS DIVA software (BD) and FlowJo software (Tree Star).

#### Membrane potential analysis

Organoids were prepared as single-cell suspension through sequential incubation with 0.25% Trypsin/EDTA and a 10 mg/ml collagenase HBSS+/+ mixture. The cells were then resuspended in plating medium (RPMI/B27, 20% knock out serum, 10 mM ROCK inhibitor Y27632) and plated onto glass-bottom cell culture dish (MatTek Corporation, P35GC1.514 C.S). The membrane potential was indicated by staining with FluoVolt™ Membrane Potential Dye (Invitrogen, F10488) at room temperature for 30 min. After wash twice with RPMI, live-cell imaging was captured with fast-frame rate video recording (2048*2048, 25 fps) on an inverted microscopy (Nikon Ti-E with Hamamatsu ORCA flash 4.0 CCD). The dynamics of the action potential traces were analyzed with a customized MATLAB algorithm.

#### Calcium transient analysis

Calcium transients were detected in plated cells from heart organoids by staining with CalciFluor™ Rhod-4, AM (Santa Cruz Biotechnology, sc-362569A). Dynamic fluorescence changes were recorded at 25 frames per second on an inverted fluorescence microscope (Nikon Ti-E with Hamamatsu ORCA flash 4.0 CCD). Data analysis of fluorescence recordings was performed in ImageJ and MATLAB. while Normalized fluorescence change ΔF/F0 was used to indicate the transient amplitude, while F_0_ indicate the fluorescence intensity at the resting status and ΔF represents the change of the fluorescence.

#### Statistics and Reproducibility

Data are presented as the mean ± standard error of the mean (SEM) for at least three replicate samples (see figure legends for additional information). Statistical significance between two groups was determined using a Student’s t-test for all quantification except RNA-seq data. Results were considered statistically significant when the *P* value was <0.05 (*P < 0.05, **P < 0.01, ***P < 0.001, ****P < 0.0001). Box plots and bar plots were generated by Prism GraphPad.

##### Binomial test

In order to test the significance of the zone predictions increased percentage of disagreement with RA treatment in the mutant cells when compared to wild-type cells, we used a binomial test. The binomial test was performed using the R function binom.test where the number of successes was the number of mutant RA- cells predicted as Ventricular (404), the number of trials was the number of mutant RA- cells (1483), and the null hypothesis probability was the percent of WTC RA- cells that were predicted as Ventricular (67.4%).

##### Wilcox *P*-values

Wilcox p-values for Figs. 2Eii, 4Dii, 5F, 7D, and 9D were calculated using the R function compare_means with p.adjust.method=bonferroni. Tables with exact p-values and sample numbers are provided as source data files.

### Reporting summary

Further information on research design is available in the Nature Research Reporting Summary linked to this article.

## Supplementary information

Supplementary Information

Description of Additional Supplementary Files

supplementary data1

supplementary data2

supplementary data3

supplementary data4

supplementary data5

supplementary data6

supplementary data7

supplementary data8

supplementary data9

supplementary data10

Supplementary Video 1

Supplementary Video 2

Supplementary Video 3

Supplementary Video 4

Supplementary Video 5

Supplementary Video 6

Reporting Summary

## Data Availability

Sequencing data underlying this study has been deposited at the Gene Expression Omnibus (GEO) database (GSE163619). Other data is available from the authors upon request.

## Code availability

Computer code used to generate results reported in this study is available on Zenodo under DIO 10.5281/zenodo.6326400. The packages used in our model and plotting code are as follows: Batchelor 1.2.4, circlize 0.4.13, clusterProfiler 3.14.3, ComplexHeatmap 2.2.0, DOSE 3.12.0, ggalluvial 0.12.3, ggplot2 3.3.5, Matrix 1.2-17, openxlsx 4.2.5, org.Hs.eg.db 3.10.0, pdp 0.7.0, Polychrome 1.3.1, preprocessCore 1.48.0, ranger 0.13.1, readr 2.1.1, rhdf5 2.30.1, scales 1.1.1, scater 1.14.6, scMerge 1.2.0, scran 1.14.6, SingleCellExperiment 1.8.0, stringr 1.4.0.
