# Supplementary material for: Computational profiling of hiPSC-derived heart organoids reveals chamber defects associated with NKX2-5 deficiency
Source: Commun Biol. 2022 Apr 29;5:399. doi: 10.1038/s42003-022-03346-4 (PMC9054831; doi:10.1038/s42003-022-03346-4)
Supplement: Supplementary file 1 — Supplementary Information [file 42003_2022_3346_MOESM1_ESM.pdf]

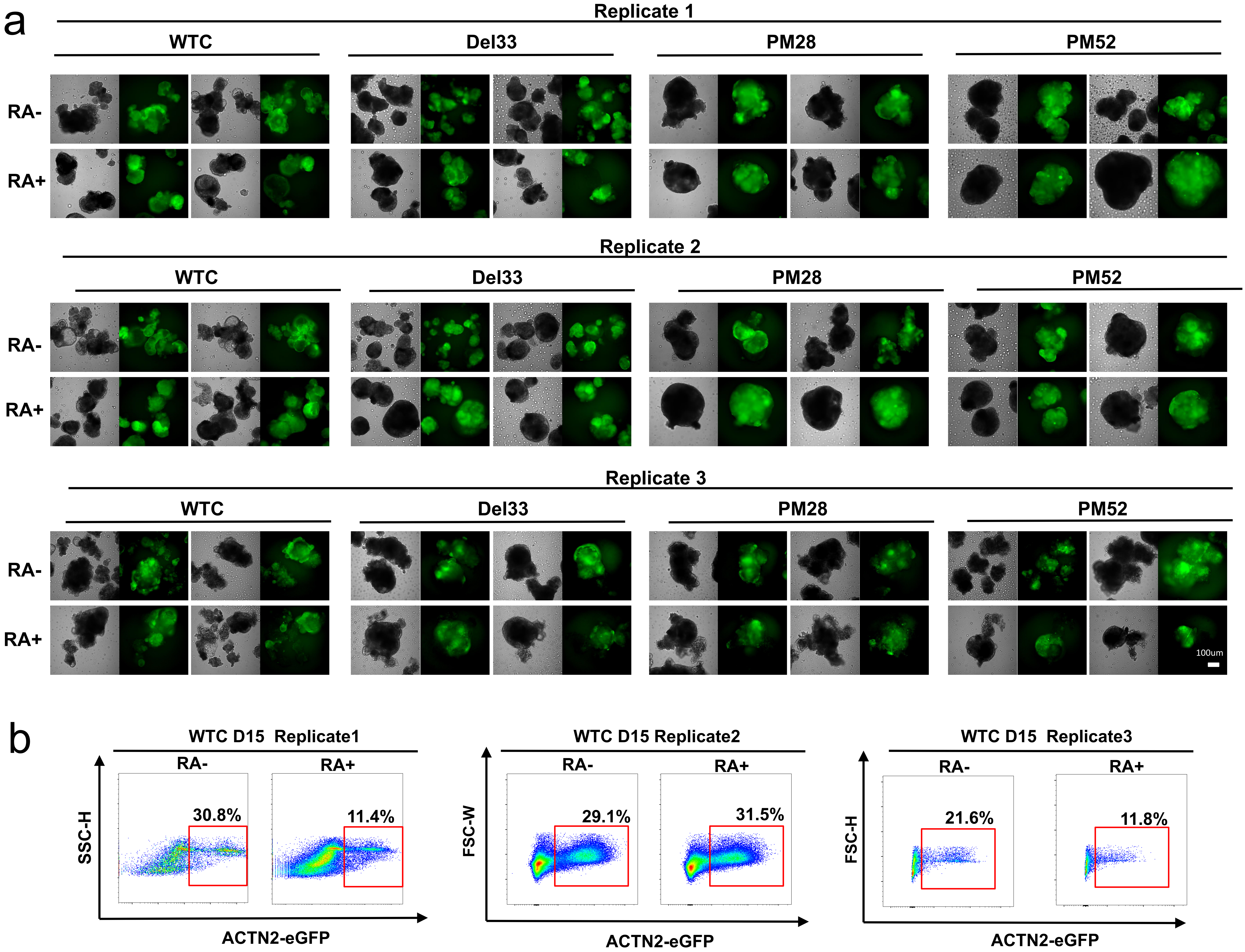

Supplementary Figure 1 Differentiation of wildtype and Nkx2-5 mutant hiPSC derived RA- and RA+ heart organoids. (**a**) Representative images of day 15 control and mutant RA- and RA+ heart organoids from three batches of differentiation. Differentiated cardiac cells were indicated by ACTN2-eGFP signal. Scale bar=100um. (**b**) Flow cytometry quantification of ACTN2-eGFP positive cells in WTC derived RA- and RA+ organoid. WTC: WTC line with ACTN2-eGFP reporter, Del33: the WTC with the first exon deletion of NKX2-5; PM28: an isogeneic line carrying NKX2-5 c.673C>A variant.

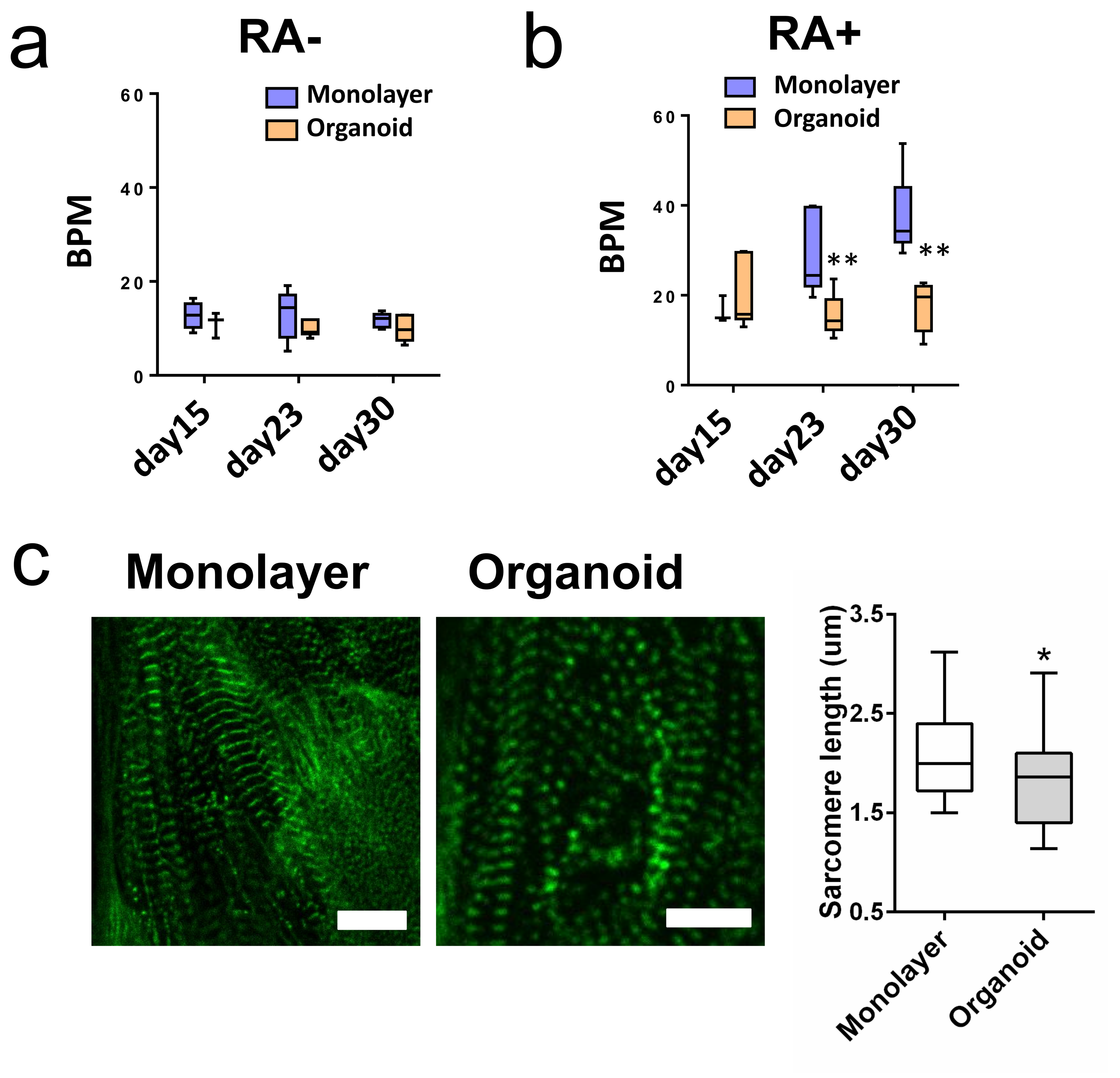

Supplementary Figure 2 Analysis of the RA- and RA+ organoids. (**a, b**) The beating rates of heart organoid and monolayer cells differentiated without RA (**a**) or under RA treatment (**b**). Data displayed as mean ± standard error, RA- day15 monolayer n=9; RA- day15 organoid n=3; RA- day23 monolayer n=6; RA- day23 organoid n=16; RA- day30 monolayer n=12; RA- day30 organoid n=13; RA+ day15 monolayer n=3; RA+ day15 organoid n=8; RA+ day23 monolayer n=5; RA+ day23 organoid n=13; RA+ day30 monolayer n=5; RA+ day30 organoid n=8. **P < 0.01, student t test. (**c**) The sarcomere alignment and length in RA- CMs from monolayer and organoid differentiations. Data displayed as mean ± standard error, n = 36 sarcomeres in each group. *P < 0.05, student t test.

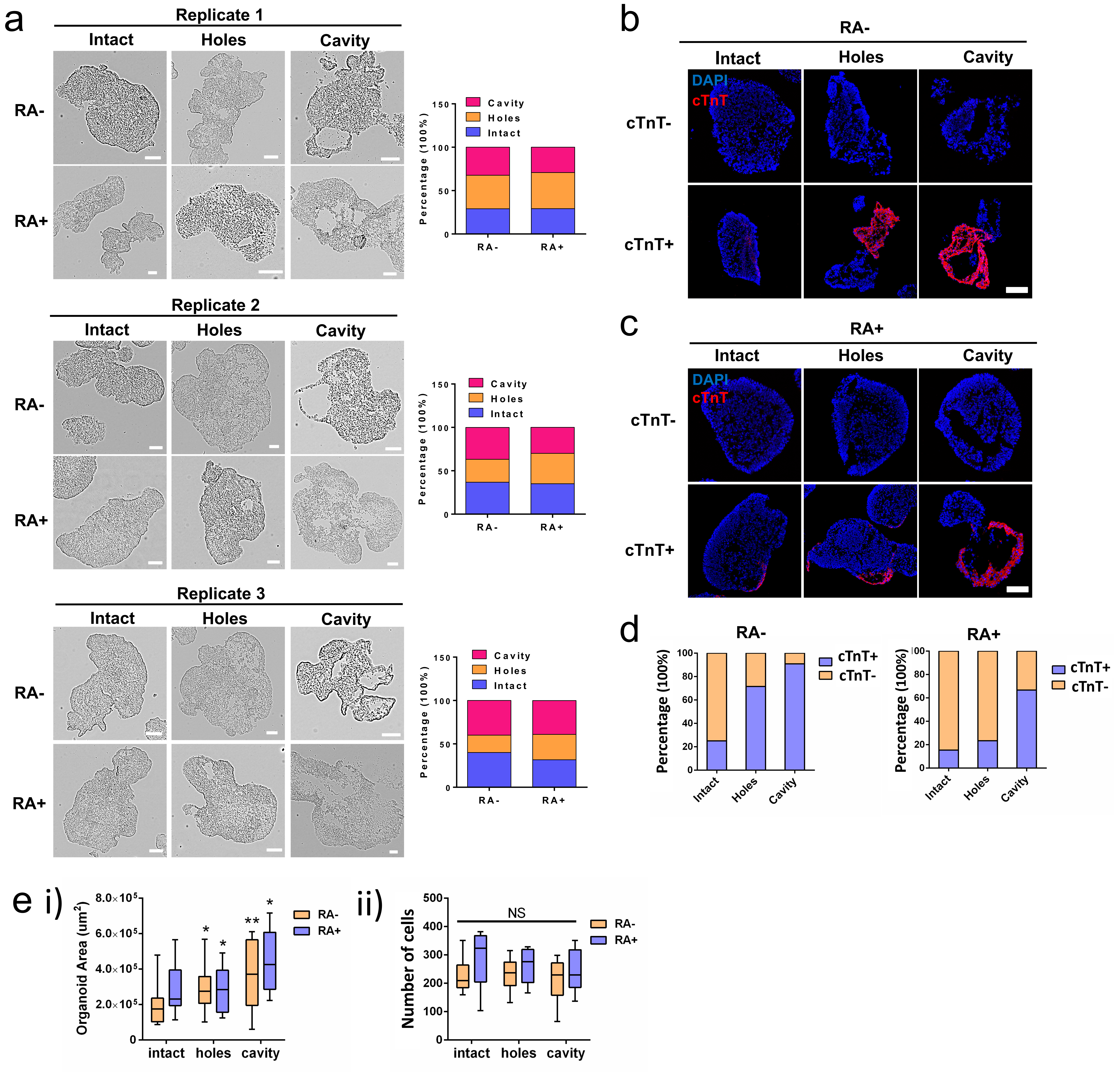

Supplementary Figure 3 Characterization of internal structures in heart organoid. (**a**) Representative images and quantification of the three organoid categories in three times of differentiations. Scale bar=100um. (**b, c, d**) The cTnT positive and negative organoids from the ventricular and atrial differentiation conditions (n=44 for RA- organoids, n=93 for RA+ organoids). scale bar=100um. (**e**) Quantification of the organoid size and cell numbers in each organoid category. Data displayed as mean ± standard error, n = 12 organoids in each group for organoid area, n=10 in each group for number of cells. *P < 0.05, ** P < 0.01, ns. not significant, student t test against Intact.

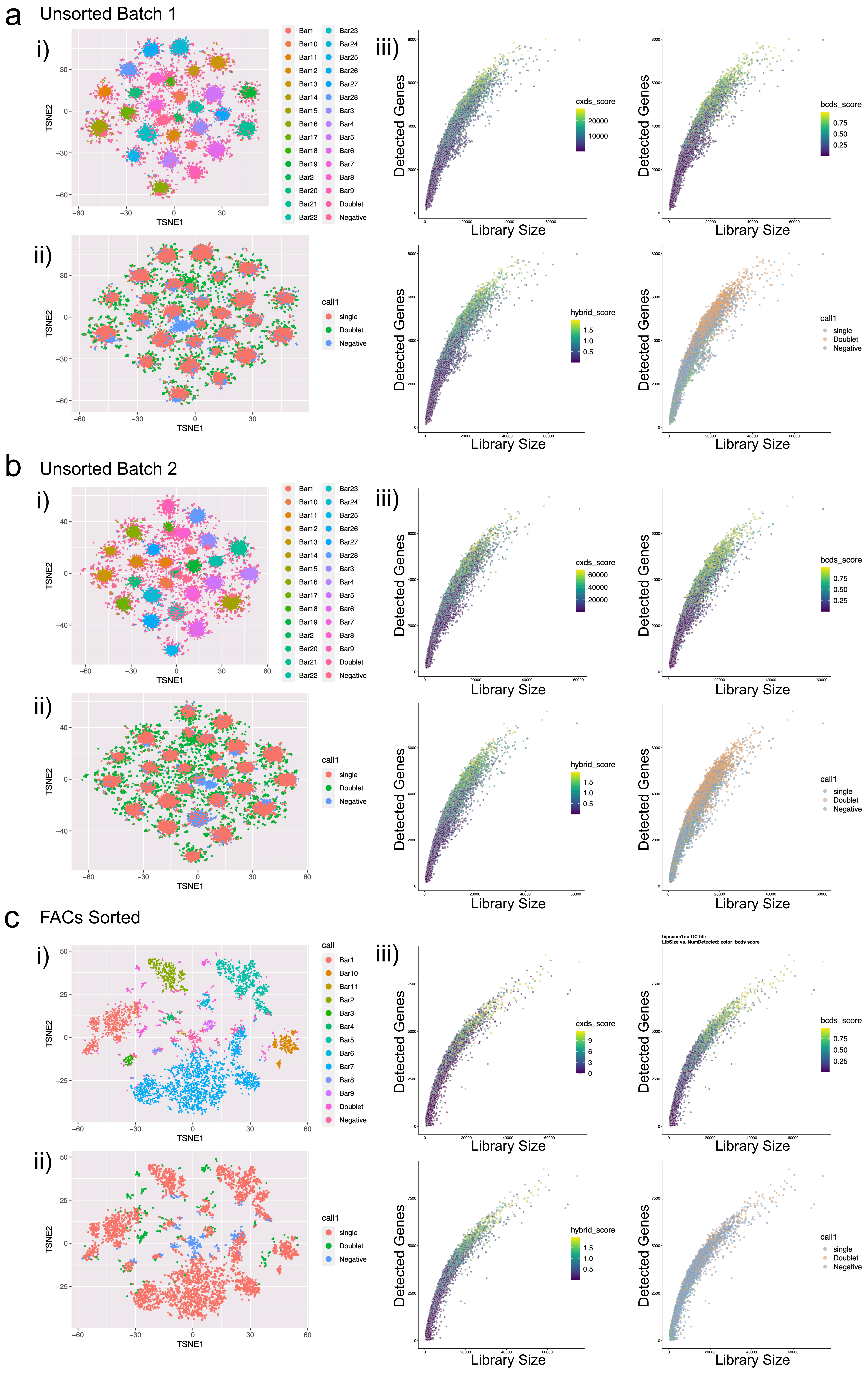
a Unsorted Batch 1
i)
ii)
iii)
b Unsorted Batch 2
c FACs Sorted
TSNE1
TSNE2
call1
single
Doublet
Negative
Bar1
Bar10
Bar11
Bar12
Bar13
Bar14
Bar15
Bar16
Bar17
Bar18
Bar19
Bar2
Bar20
Bar21
Bar22
Bar23
Bar24
Bar25
Bar26
Bar27
Bar28
Bar3
Bar4
Bar5
Bar6
Bar7
Bar8
Bar9
call
Detected Genes
Library Size
cxds_score
bcds_score
hybrid_score
hipsccm1no QC filt:
LibSize vs. NumDetected; color: bcds score

Supplementary Figure 4 Analysis_of the MULTI-seq barcodes and doublet scores in the three batches of scRNA-seq. (**a-c**) i) TSNE projection of single cells using barcodes as features grouped by i) barcodes and ii) grouped by singlets, doublets, or negatives based on their expression of barcodes. iii) The doublet scores generated by scds along with doublet call in order to remove the doublets that failed to be identified by barcodes.

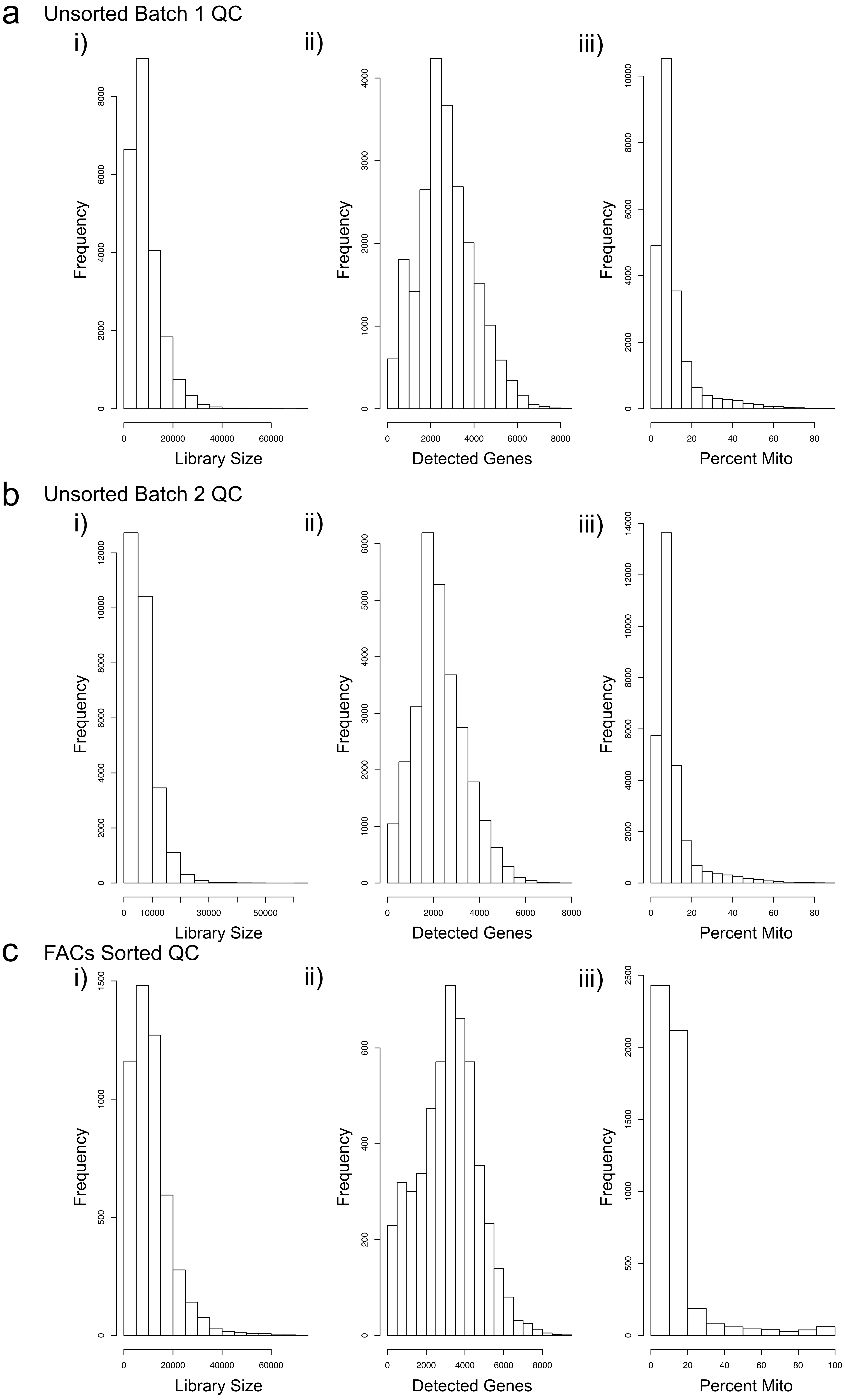

Supplementary Figure 5 Distribution plot of three parameters used to identify the low quality cells. (**a-c**) The plot of i) library sizes, ii) total gene numbers, and iii) mitochondria percentages in three batches of experiments.

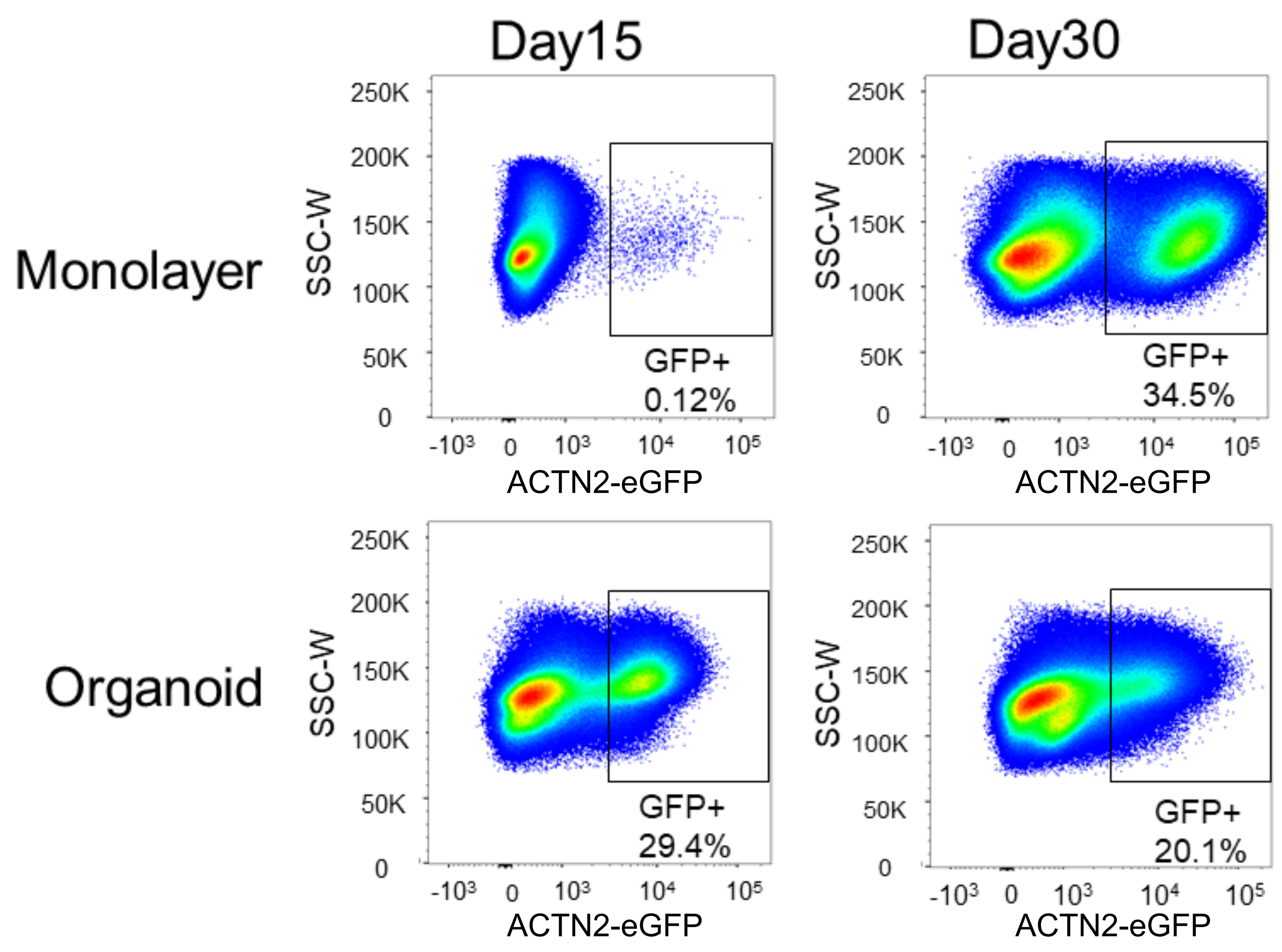

Supplementary Figure 6 Quantification and isolation of GFP positive cells from monolayer and organoid differentiation at day 15 and 30.

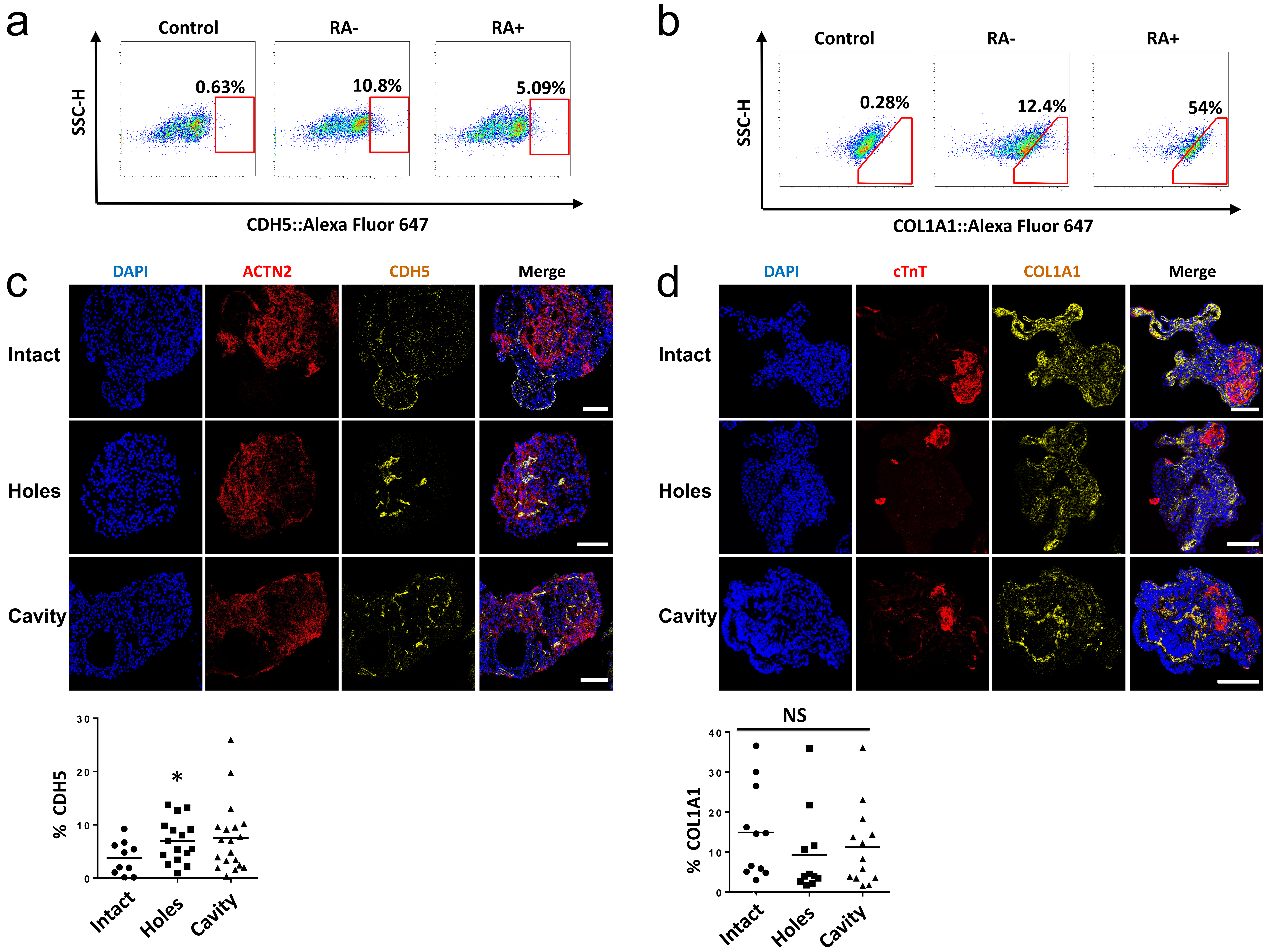

Supplementary Figure 7 Quantification of the endothelial cell and fibroblast in heart organoids. (**a, b**) Flow cytometry analysis of endothelial cell and fibroblast in RA- and RA+ WTC organoids. Note the controls are hiPSCs representing negative controls. (**c, d**) Immunostaining analysis of CDH5 and COL1A1 in each organoid category. The percentages were calculated using the marker and DAPI staining areas. (Figure c: n=10 for intact, n=16 for holes, n=17 for cavity; Figure d: n=11 for intact and holes, n=13 for cavity) *$P < 0.05$, ns. not significant, student t test against Intact.

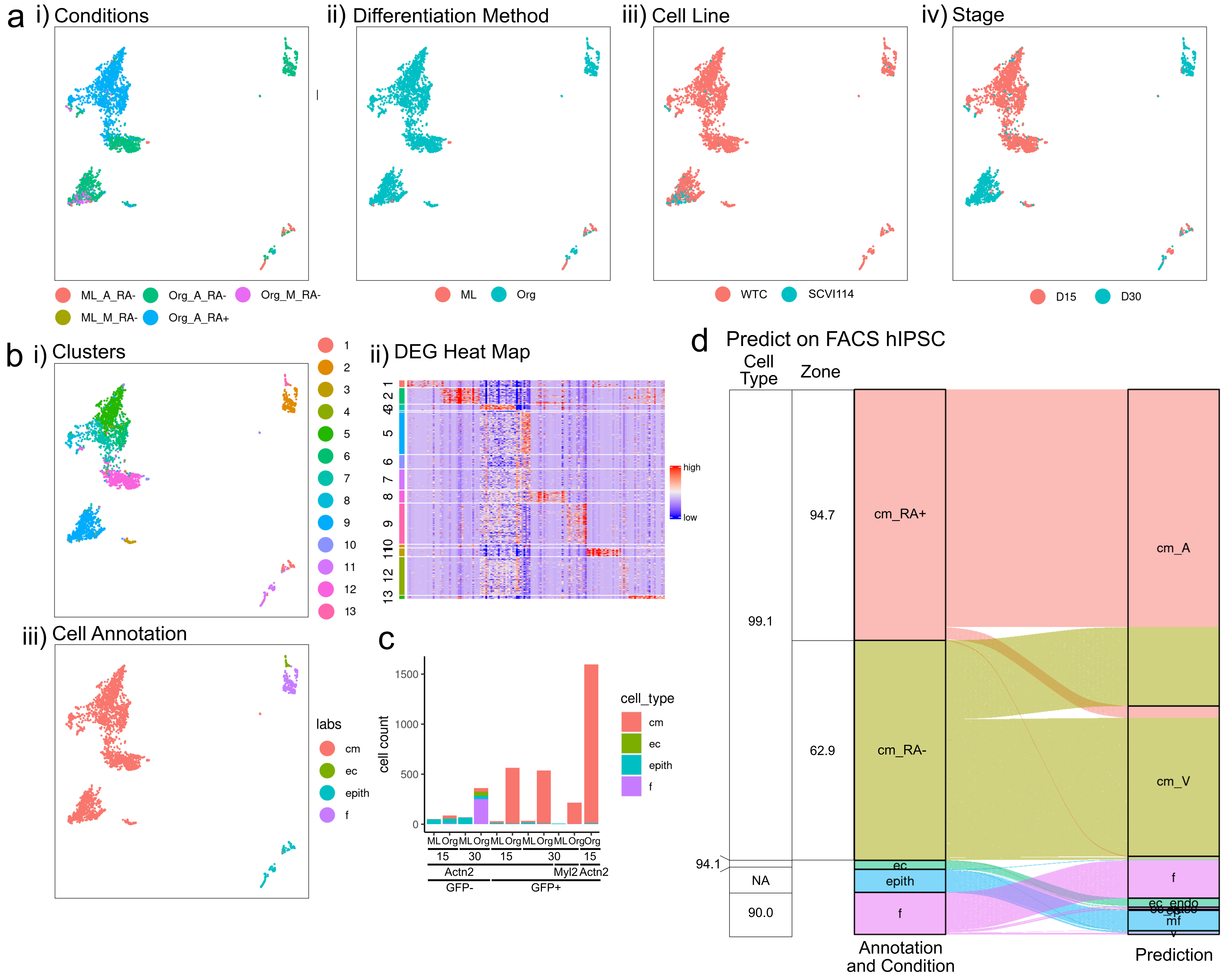

Supplementary Figure 8 Prediction of FACs sorted RA- and RA+ cells using the validated hierarchal random forest model. (**a**) UMAP projections of RA- and RA+ single cells grouped by i) conditions, ii) differentiation methods, iii) cell lines, and iv) stages. (**b**) Unsupervised clustering of the single cells. i) UMAP projection of the clusters and ii) cluster-specific differentially expressed genes with cluster labels. iii) UMAP projection of single cells grouped by cell type (**c**) The number of profiled cells in each condition colored proportionately by annotated cell type. (**d**) Sankey diagram of prediction results of wild type RA- and RA+ cells (Anomalies removed). Table provides cell type and conditional zone accuracies. ML: monolayer, Org: organoid, A: WTC line with ACTN2-eGFP reporter, M: WTC line with MYL2-eGFP reporter, cm: cardiomyocyte, ec: endothelial cell, epith: epithelial cell, ec_endo: endocardial endothelial cell, f: fibroblasts, mf: macrophage, v: vascular.

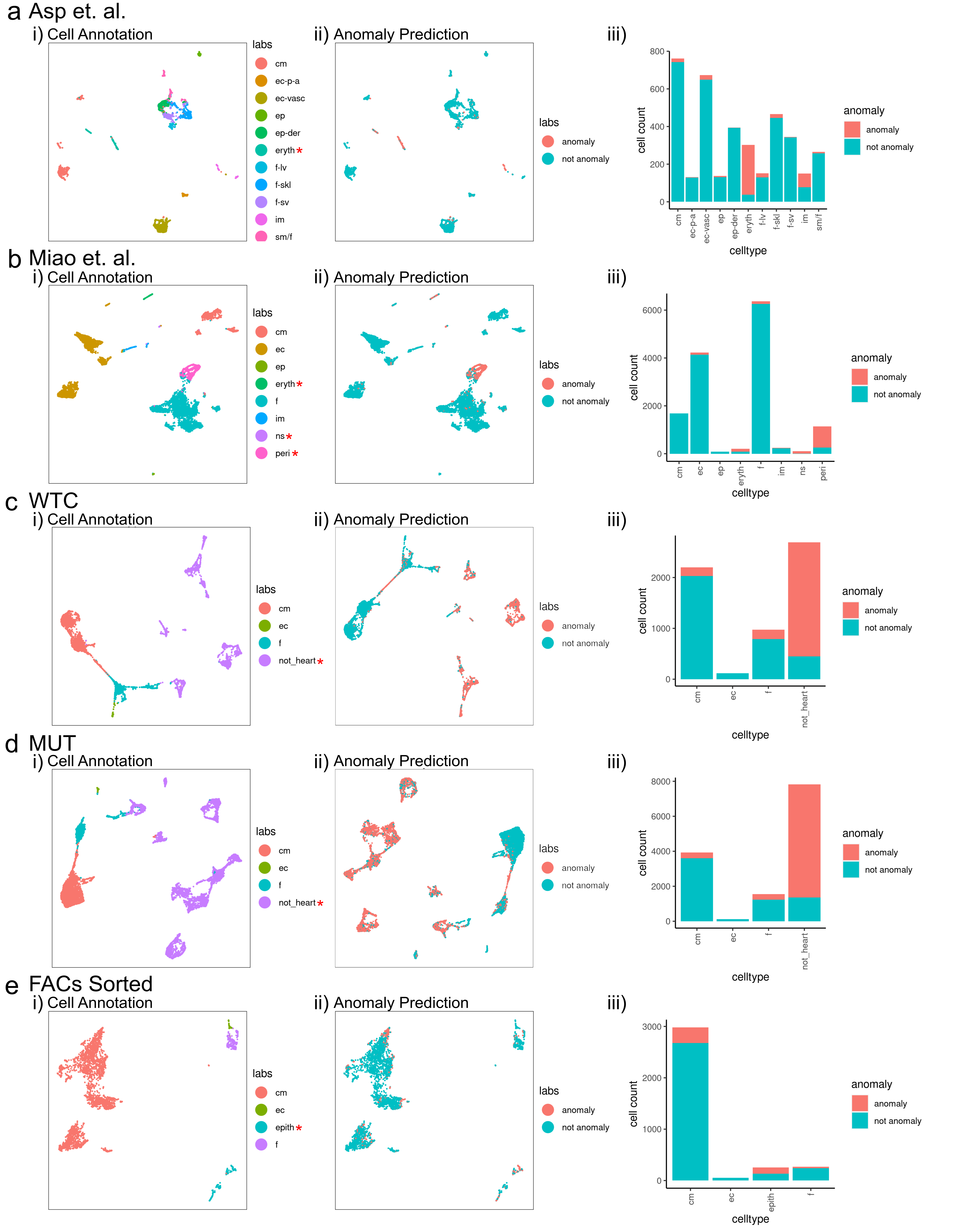
a Asp et. al.
i) Cell Annotation
labs
cm
ec-p-a
ec-vasc
ep
ep-der
eryth *
f-lv
f-skl
f-sv
im
sm/f
ii) Anomaly Prediction
labs
anomaly
not anomaly
iii)
cell count
0
200
400
600
800
cm
ec-p-a
ec-vasc
ep
ep-der
eryth
f-lv
f-skl
f-sv
im
sm/f
celltype
anomaly
anomaly
not anomaly
b Miao et. al.
i) Cell Annotation
labs
cm
ec
ep
eryth *
f
im
ns *
peri *
ii) Anomaly Prediction
labs
anomaly
not anomaly
iii)
cell count
0
2000
4000
6000
cm
ec
ep
eryth
f
im
ns
peri
celltype
anomaly
anomaly
not anomaly
c WTC
i) Cell Annotation
labs
cm
ec
f
not_heart *
ii) Anomaly Prediction
labs
anomaly
not anomaly
iii)
cell count
0
1000
2000
cm
ec
f
not_heart
celltype
anomaly
anomaly
not anomaly
d MUT
i) Cell Annotation
labs
cm
ec
f
not_heart *
ii) Anomaly Prediction
labs
anomaly
not anomaly
iii)
cell count
0
2000
4000
6000
8000
cm
ec
f
not_heart
celltype
anomaly
anomaly
not anomaly
e FACs Sorted
i) Cell Annotation
labs
cm
ec
epith *
f
ii) Anomaly Prediction
labs
anomaly
not anomaly
iii)
cell count
0
1000
2000
3000
cm
ec
epith
f
celltype
anomaly
anomaly
not anomaly

Supplementary Figure 9 | Anomaly prediction on the scRNA-seq datasets used in the study. (**a-e**) i) UMAP projections of single cells grouped by cell types. The star indicates which cell types should be identified as Anomalies. ii) UMAP projections of single cells grouped by Anomaly prediction. iii) The number of cells in each cell type colored proportionately by the Anomaly prediction. Abbreviations: cm = cardiomyocytes, ep = epicardial cell, f = fibroblast, ec_vasc = vascular endothelial cells, ec_endo = endocardial endothelial cells, v = valvar, mf = macrophage, b/t = b/t cell, 5w = undifferentiated cells, cm_myzo = Myoz2-enriched CMs, f-lv = fibroblast-like: (related to larger vascular development), f-sv = fibroblast-like: (related to smaller vascular development), sm/f = Smooth muscle cells / fibroblast-like), f-skl = Fibroblast-like: (related to cardiac skeleton connective tissue), ep-der = Epicardium-derived cells, ec-p-a = Endothelium / pericytes / adventitia, im = immune cells, eryth = erythrocytes, peri = pericyte, ns = nervous system

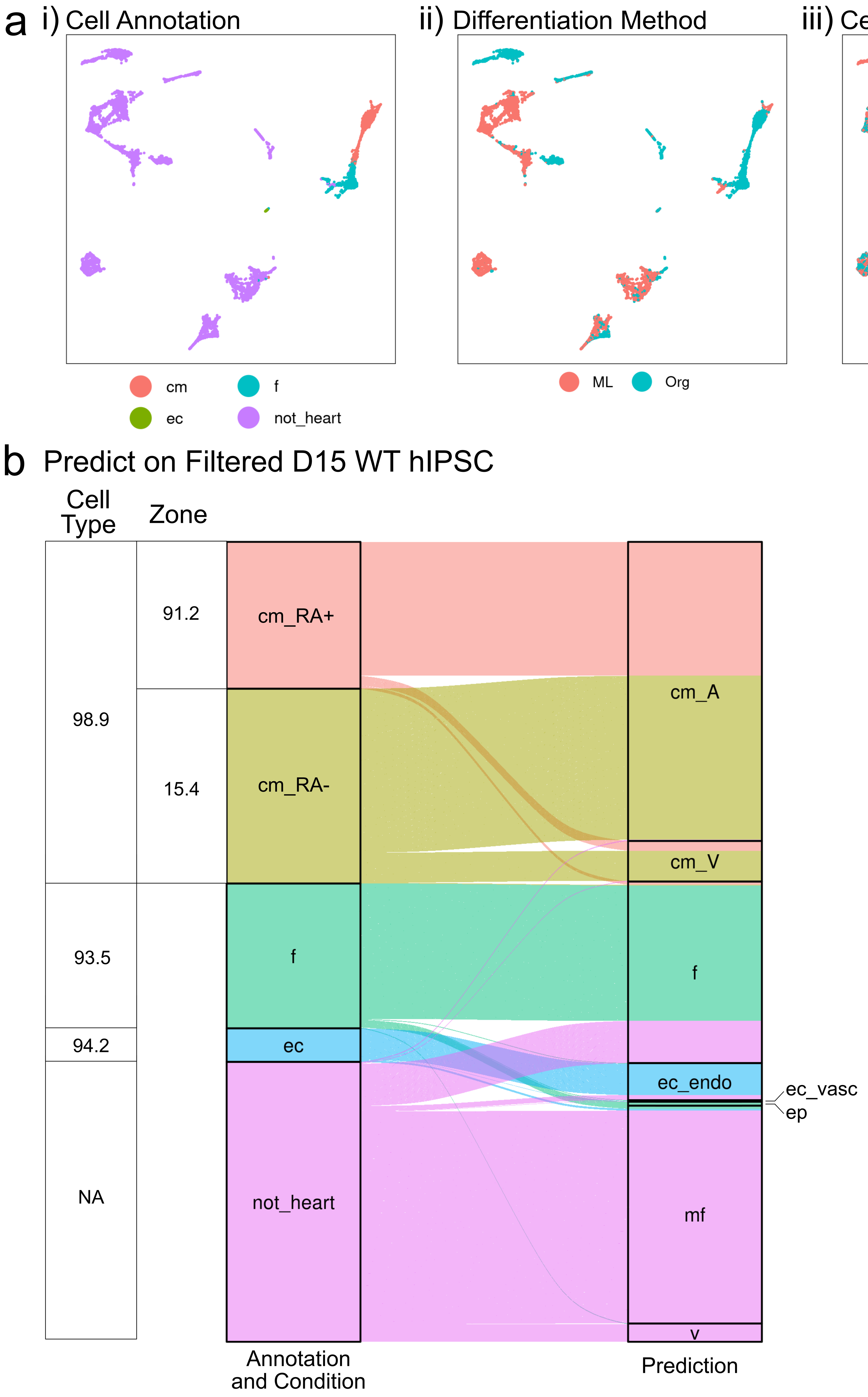

Supplementary Figure 10 Analysis of the wild type RA- and RA+ cells at day 15. (**a**) UMAP projections of RA- and RA+ single cells grouped by i) cell annotations, ii) differentiation methods, iii) cell lines, and iv) treatment. (**b**) Sankey diagram of prediction results of D15 wild type RA- and RA+ cells (Anomalies removed). Table provides cell type and conditional zone accuracies. ML: Monolayer, Org: organoid, WTC: WTC line with ACTN2-eGFP reporter, cm: cardiomyocyte, ec: endothelial cell, f: fibroblasts, ep: epithelial cell, ec_vasc: vascular endothelial cell, ec_endo: endocardial endothelial cell, mf: macrophage, v: vascular

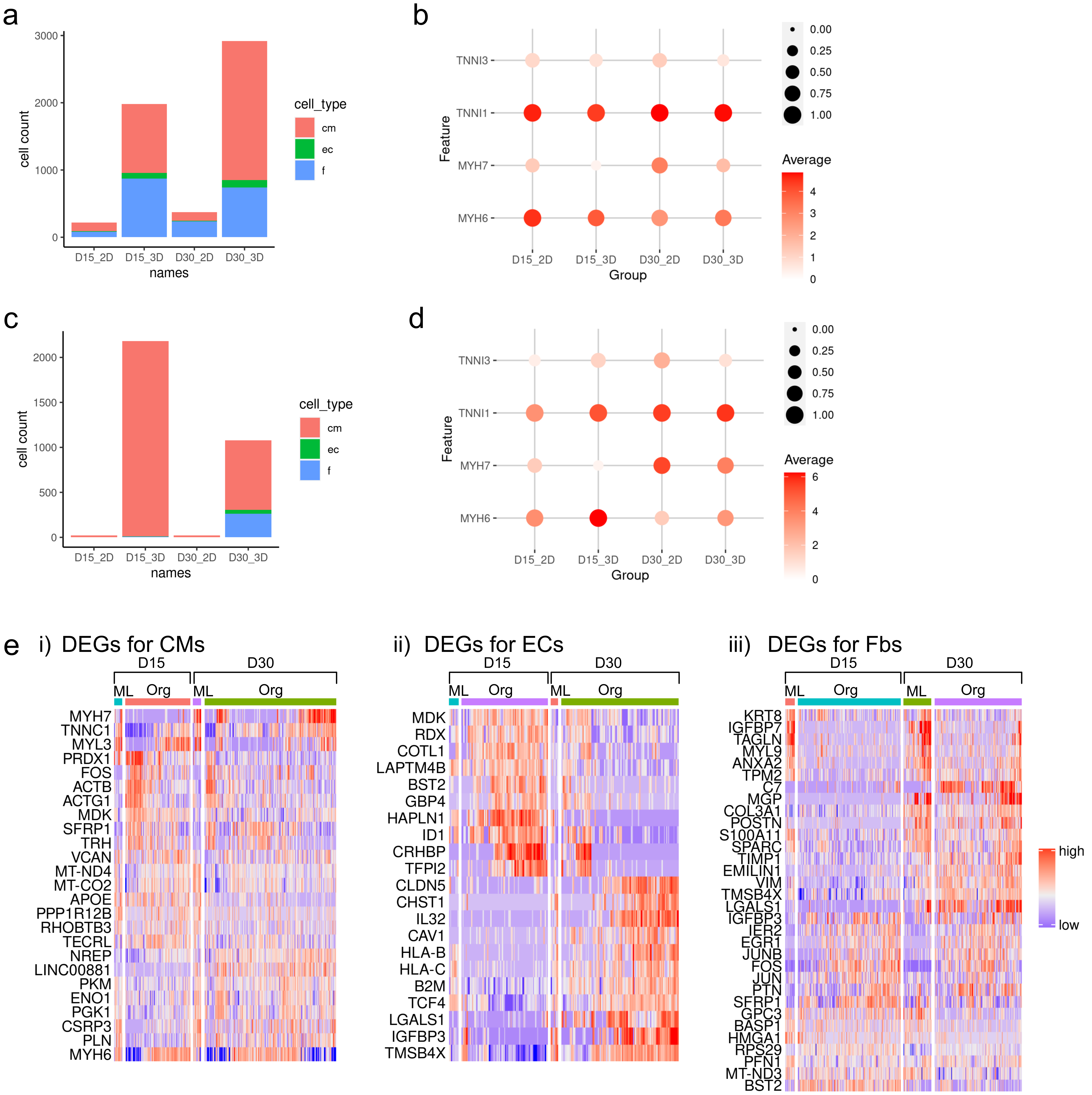

Supplementary Figure 11 Comparative analysis of the transcriptional profiles at different stages. (**a,c**)The number of cells profiled at each stage colored proportionately by their annotated cell type. (**b,d**) Dot plots of the expression of maturation genes at different stages and differentiation systems. (**e**) Heatmaps of differentially expressed genes (Supplementary Data 4) between different conditions and stages in each cardiac cell type. D15: day15, D30: day 30, 2D: monolayer, 3D: organoid, ML: monolayer, Org: organoid.

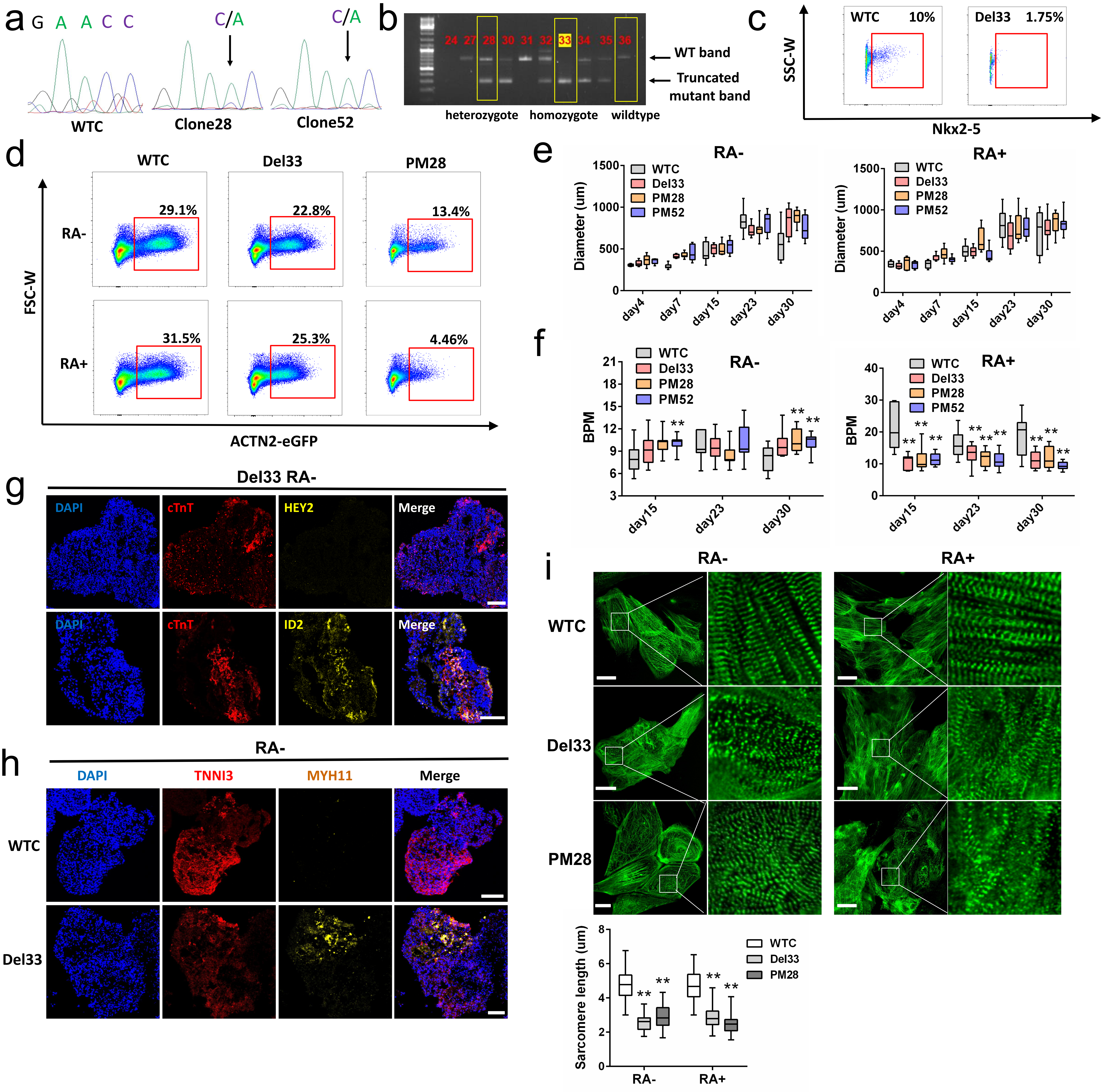

Supplementary Figure 12 Identification of functional and structural defects in mutant organoids. (**a,b**) PCR and sanger sequencing verification of single nucleotide mutation in PM28 and PM52 isogenic lines (a) and first exon deletion in Del33 line (**b**). (**c**) Flow cytometry analysis of Nkx2-5 expression in control and Del33 hiPSC derived cardiomyocytes from Day7 of differentiation. (**d**) Reduced cardiomyocyte differentiation efficiency in mutant organoids were identified by flow cytometry based on the ACTN2-eGFP signal. (**e**) The diameters in wild type and mutant RA- and RA+ organoids at day 30 (day4, n=5 organoids; day7, n=9 organoids; day15 and day30, n=10 organoids; day23, n=12 organoids). (**f**) The beating rates in wild type and mutant RA- and RA+ organoids at day 30. n=10 organoids, ** P < 0.01, student t test against WTC. (**g**) Immunostaining analysis of HEY2 and ID2 expression in Del33 RA- organoids on differentiation day30. Scale bar=100um. (**h**) Ectopic expression of MYH11 was found in Del33 RA- organoids on day30. Scale bar=100um. (**i**) Defective sarcomere structures were identified in mutant CMs replated from heart organoids. Scale bar=20um. WTC RA- N=180 sarcomeres; WTC RA+ N=152 sarcomeres; Del33 RA- N=84 sarcomeres; Del33 RA+ N=157 sarcomeres; PM28 RA- N=159 sarcomeres; PM28 RA+ N=81 sarcomeres, data displayed as mean ± standard error. ** P < 0.01, student t test against WTC.

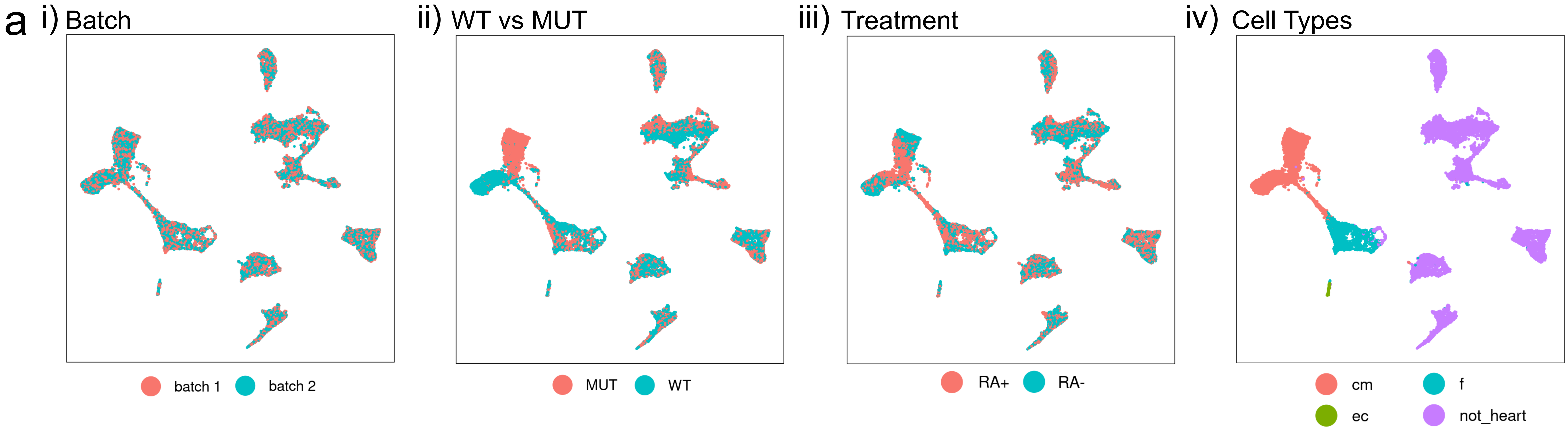

Supplementary Figure 13 UMAP projections of unsorted hiPSC cells grouped by (i) batch, (ii) wild type vs mutant cell lines, (iii) treatment, and (iv) annotated cell types.

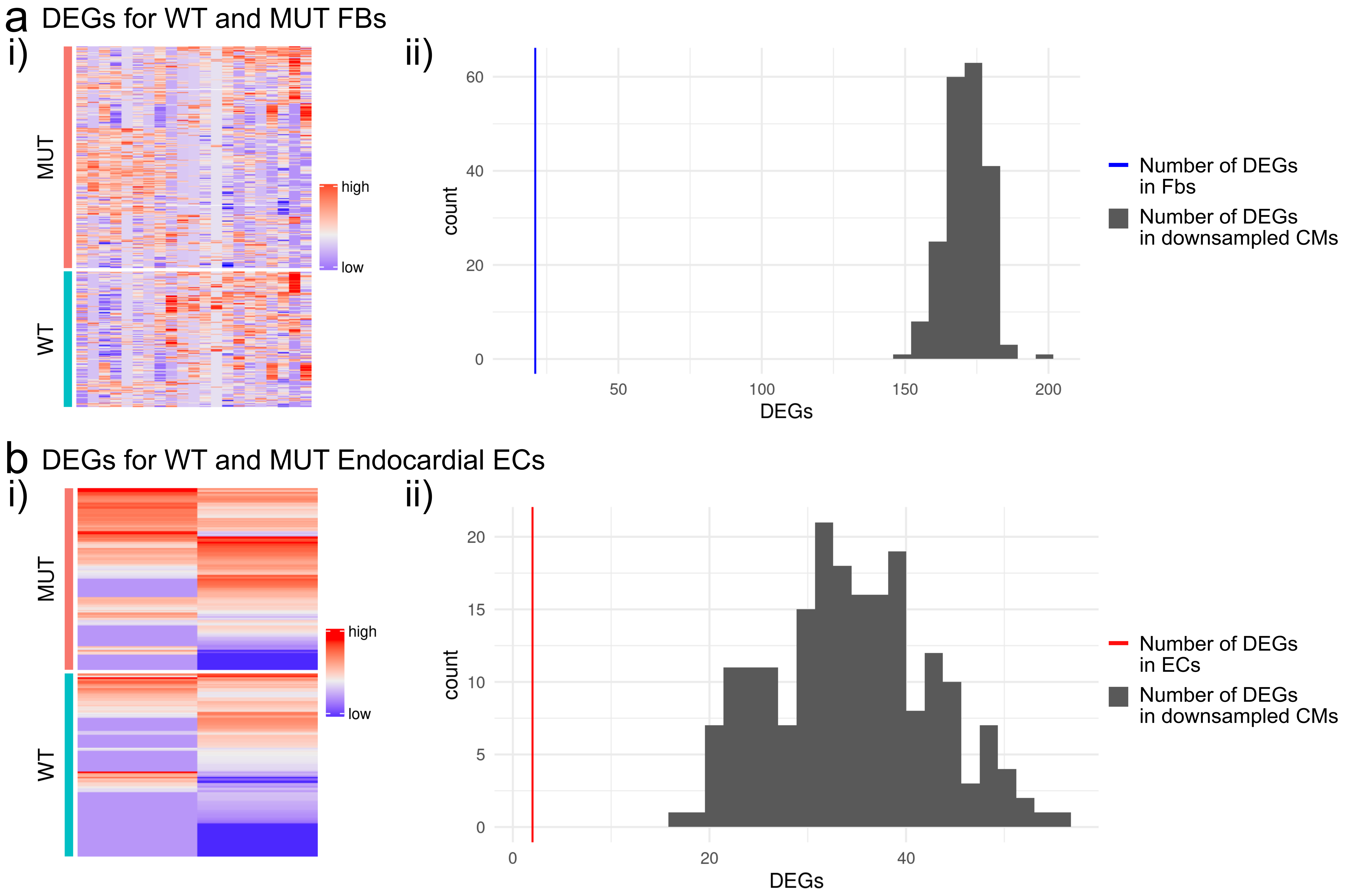

Supplementary Figure 14 Differentially expressed genes between wild type and mutant cell lines in predicted fibroblasts (FB) and endocardial endothelial cells (EC). (**ai**) Heatmap of differentially expressed genes (DEGs) between wild type and mutant cell lines in predicted FBs. (**aii**) Histogram of the number of DEGs found (grey) for predicted CMs when repetitively down sampled 200 times compared to the number of FB DEGs (blue). Each time, CMs were down sampled to 2,158 cells to match the number of predicted FBs and DEGs were recalculated. (**bi**) Heatmap of DEGs between wild type and mutant cell lines in EndoECs. (**bii**) Histogram of the number of DEGs found (grey) for predicted CMs when repetitively down sampled 200 times compared to the number of EndoEC DEGs (red). Each time, CMs were down sampled to 279 cells to match the number of predicted EndoECs and DEGs were recalculated.

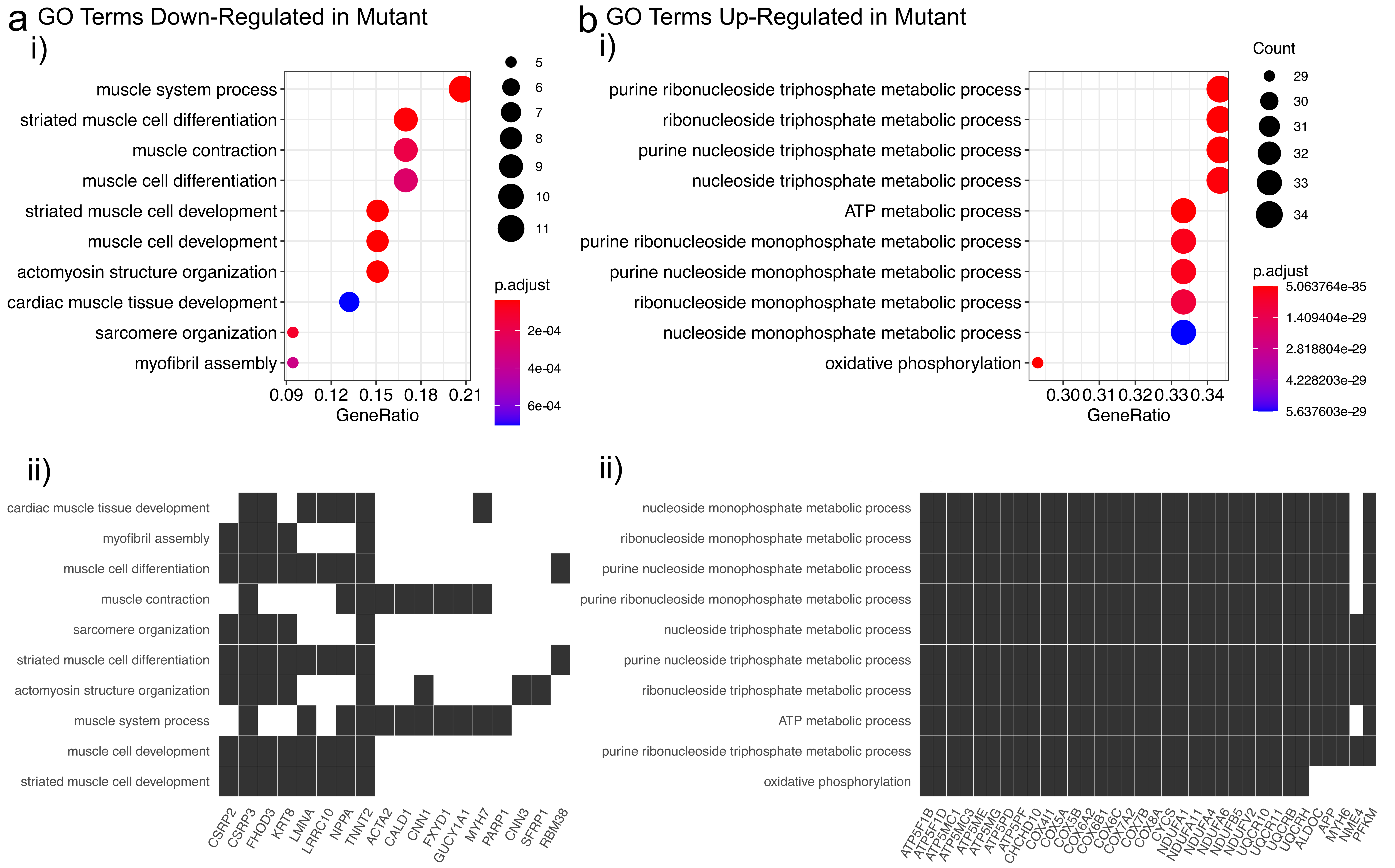

Supplementary Figure 15 GO Analysis of predicted cardiomyocytes in the unsorted hiPSC dataset. (**a**) GO terms down regulated in mutant cell lines. (i) Dot plot of top ten down regulated GO terms colored by adjusted p-value. (ii) Chart depicting which down regulated genes contribute to the top GO terms. (**b**) GO terms up regulated in mutant cell lines. (i) Dot plot of top ten up regulated GO terms colored by adjusted p-value. (ii) Chart depicting which up regulated genes contribute to the top GO terms.

a

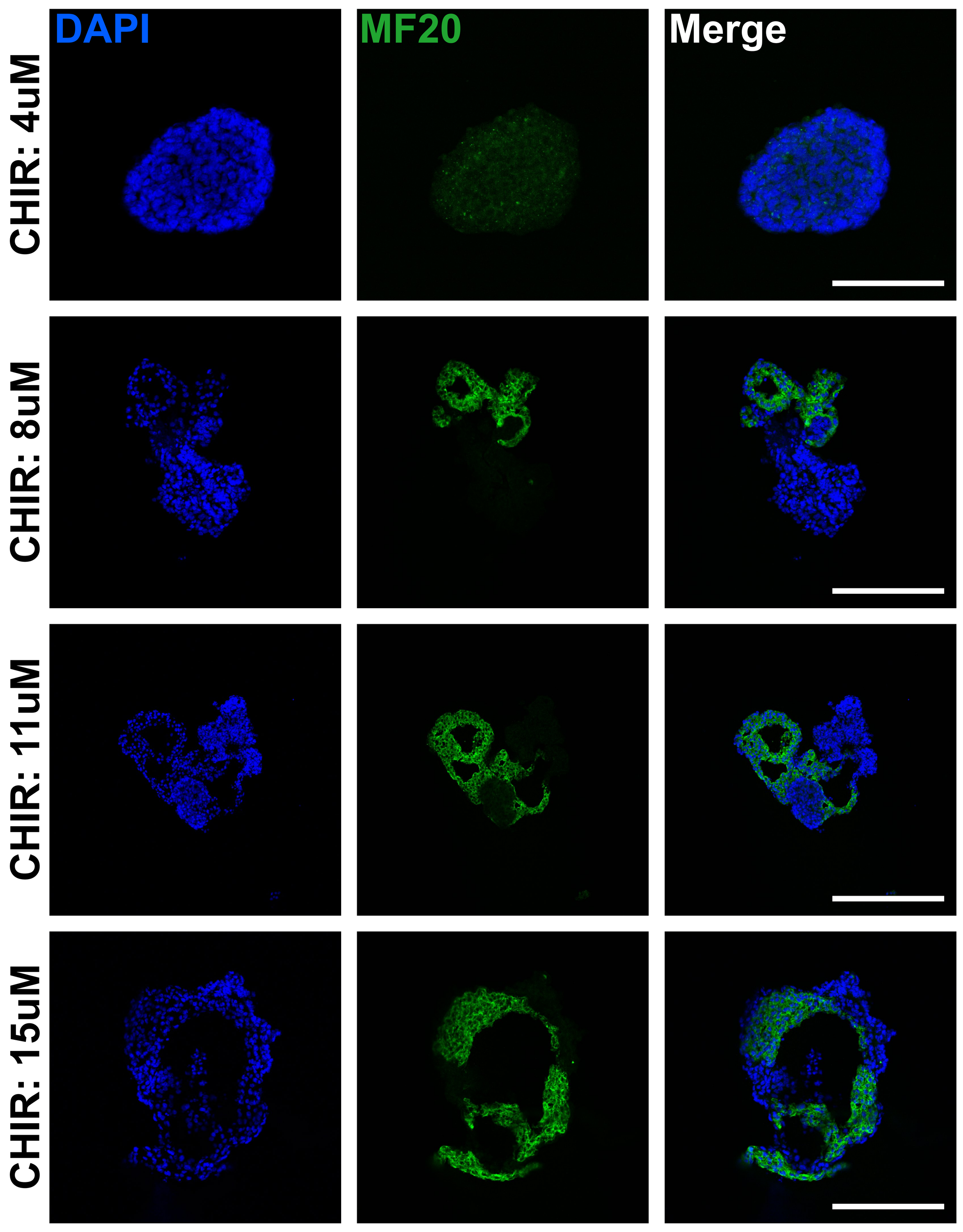

b

		Categories			
		Total	Intact	Holes	Cavity
CHIR	4 µM	2	2	0	0
	8 µM	14	8	5	1
	11 µM	22	6	15	1
	15 µM	20	0	10	10

Supplementary Figure 16 Wnt signaling affects organoid structure. (**a**) Representative Organoids at differentiation day 14 stained with MF20 and DAPI after being treated with different CHIR concentrations. (**b**) The number of organoids analyzed in each group and their respective categories (intact, holes, cavity). A transition from mainly intact organoids to organoids with holes and cavity was observed as the CHIR concentration increased. scale bar=100um

PMID:30464173				
	cm	ec	f	not_heart
cm	257	0	0	6
other	2	16	318	6787

Supplementary Table 1 SingleR Label Transfer results obtained from Churko et al. (PMID: 30464173) The rows represent the labels on the Churko et al. dataset while the columns represent the labels on our monolayer dataset. cm= cardiomyocyte, ec=endothelial cells, f=fibroblasts, not_heart=non-cardiac cells, other=non-cardiomyocytes from Churko et al. dataset.

PMID:30290179				
	cm	ec	f	not_heart
cm	258	0	2	26
other	1	16	316	6767

Supplementary Table 2 SingleR Label Transfer results obtained from Friedman et al. (PMID: 30290179). The rows represent the labels on the Friedman et al. dataset while the columns represent the labels on our monolayer dataset. cm= cardiomyocyte, ec=endothelial cells, f=fibroblasts, not_heart=non-cardiac cells, other=non-cardiomyocytes from Friedman et al. dataset.

sgRNAs to generate Nkx2-5 loss of function mutants	
HNKX2-5- deletion-up-sgRNA-F1	GCCAGTCTCACAGCGCCAGG
HNKX2-5- deletion-up-sgRNA-R1	CCTGGCGCTGTGAGACTGGC
HNKX2-5-deletion-down-sgRNA-F3	GGGGACGAAAGCGACCCAGG
HNKX2-5-deletion-down-sgRNA-R3	CCTGGGTCGCTTTCGTCCCC
sgRNAs to introduce a single nucleotide mutation	
hNKX2-5-point-sgRNA-F3	caccgGCCGCTTGCACTTGTAGCGC
hNKX2-5-point-sgRNA-R3	aaacGCGCTACAAGTGCAAGCGGCc
ssODN-hNkx2-5_188	CCAGCTGGCCAGCGTGCTGAAACTCACGTCCACGCAGGTCAAGATCTGGTTCCAGAAACGGCGCTACAAGTGCAAGCGGCAGCGGCAGGACCAGACTCTGGAGCTGGTGGGGCTgccccc
Genotyping primers to validate Nkx2-5 first exon deletion	
HNKX2-5-deletion-G-F:	AAACTGCTCATCGCTCCTGT
HNKX2-5-deletion-G-R:	CGACAACACCAGGCATCTTA
Genotyping primers to validate a single nucleotide mutation	
hNKX2-5-point-genotyping-F	GGTGGAGCTGGAGAAGACAG
hNKX2-5-point-genotyping-R4	ATAGGCGGGGTAGGCGTTAT

Supplementary Table 3 The sgRNA , single-stranded oligodeoxynucleotide (ssODN) sequences and PCR primers used to generate hiPSC lines carrying a single nucleotide variant (c.673C>A) and first exon deletion in NKX2-5.

Anchor LMO	5'-TGGAATTCTCGGGTGCCAAGGgtaacgatccagctgtcact-Lipid-3'
Co-Anchor LMO	5'-Lipid-AGTGACAGCTGGATCGTTAC-3'
MULTI-seq Additive Primer	5'-CTTGGCACCCGAGAATTCC-3'
TruSeq RPIX Primer	5'-CAAGCAGAAGACGGCATACGAGATNNNNNNGTG ACTGGAGTT CCTTGGCACCCGAGAATTCCA-3'
TruSeq Universal Adapter Primer	5'-AATGATACGGCGACCACCGAGATCTACACTCTTTCCCTACACGA CGCTCTTCCGATCT-3'
BC1	CCTTGGCACCCGAGAATTCCAGGAGAAG AAAAAAAAAAAAAAAAA AAAAAAAAAAAAA
BC2	CCTTGGCACCCGAGAATTCCACCACAATGAAAAAAAAAAAAAAAAA AAAAAAAAAAAAA
BC3	CCTTGGCACCCGAGAATTCCATGAG ACCTAAAAAAAAAAAAAAAAA AAAAAAAAAAAAA
BC4	CCTTGGCACCCGAGAATTCCAGCACACGCAAAAAAAAAAAAAAAA AAAAAAAAAAAAAA
BC5	CCTTGGCACCCGAGAATTCCAAGAG AGAGAAAAAAAAAAAAAAAA AAAAAAAAAAAAAA
BC6	CCTTGGCACCCGAGAATTCCATCACAGCAAAAAAAAAAAAAAAAAA AAAAAAAAAAAAA
BC7	CCTTGGCACCCGAGAATTCCAGAAAAGGGAAAAAAAAAAAAAAAA AAAAAAAAAAAAAA
BC8	CCTTGGCACCCGAGAATTCCACGAGATTCAAAAAAAAAAAAAAAAA AAAAAAAAAAAAA
BC9	CCTTGGCACCCGAGAATTCCAGTAGCACTAAAAAAAAAAAAAAAAA AAAAAAAAAAAAA
BC10	CCTTGGCACCCGAGAATTCCACGACCAGCAAAAAAAAAAAAAAAA AAAAAAAAAAAAAA
BC11	CCTTGGCACCCGAGAATTCCATTAGCCAGAAAAAAAAAAAAAAAAA AAAAAAAAAAAAA
BC12	CCTTGGCACCCGAGAATTCCAGGACCCCAAAAAAAAAAAAAAAAA AAAAAAAAAAAAAA
BC13	CCTTGGCACCCGAGAATTCCACCAACCGGAAAAAAAAAAAAAAAA AAAAAAAAAAAAAA
BC14	CCTTGGCACCCGAGAATTCCATGACCGATAAAAAAAAAAAAAAAAA AAAAAAAAAAAAA
BC15	CCTTGGCACCCGAGAATTCCAGCAACGCCAAAAAAAAAAAAAAAA AAAAAAAAAAAAAA
BC16	CCTTGGCACCCGAGAATTCCACAATCGGTAAAAAAAAAAAAAAAAA AAAAAAAAAAAAA
BC17	CCTTGGCACCCGAGAATTCCAATAGCGTCAAAAAAAAAAAAAAAAA AAAAAAAAAAAAA
BC18	CCTTGGCACCCGAGAATTCCAGAATCTCGAAAAAAAAAAAAAAAAA AAAAAAAAAAAAA
BC19	CCTTGGCACCCGAGAATTCCACTAGCTGAAAAAAAAAAAAAAAAAA AAAAAAAAAAAAA

BC20	CCTTGGCACCCGAGAATTCCAAGACCTTGAAAAAAAAAAAAAAAA<br>AAAAAAAAAAAAA
BC21	CCTTGGCACCCGAGAATTCCAGAAGGAAGAAAAAAAAAAAAAAA<br>AAAAAAAAAAAAAA
BC22	CCTTGGCACCCGAGAATTCCACTACGACAAAAAAAAAAAAAAAA<br>AAAAAAAAAAAAA
BC23	CCTTGGCACCCGAGAATTCCAAGAAGAGGAAAAAAAAAAAAAAA<br>AAAAAAAAAAAAAA
BC24	CCTTGGCACCCGAGAATTCCAGTACGCATAAAAAAAAAAAAAAAA<br>AAAAAAAAAAAAA
BC25	CCTTGGCACCCGAGAATTCCACGAAGCCCAAAAAAAAAAAAAAA<br>AAAAAAAAAAAAAA
BC26	CCTTGGCACCCGAGAATTCCAACATGCGTAAAAAAAAAAAAAAAA<br>AAAAAAAAAAAAA
BC27	CCTTGGCACCCGAGAATTCCATAAGGCTCAAAAAAAAAAAAAAAA<br>AAAAAAAAAAAAA
BC28	CCTTGGCACCCGAGAATTCCAGGAAGGAAAAAAAAAAAAAAAAAA<br>AAAAAAAAAAAAAA

Supplementary Table 4 The MULTI-seq lipid indices, barcode sequences and oligonucleotides used to generate sequencing libraries.

cy5-hWt1-Right-1	GCA TGT TGT GAT GGC GGA CTT TAT ACG TCG AGT TGA ACG TCG TAA CA
cy5-hWt1-left-1	TAG CGC TAA CAA CTT ACG TCG TTA TGA ATT CAT CTG ACC GGG CAA A
cy5-hWt1-Right-2	TCT CTT ATT GCA GCC TGG GTT TAT ACG TCG AGT TGA ACG TCG TAA CA
cy5-hWt1-left-2	TAG CGC TAA CAA CTT ACG TCG TTA TGA AGC ACA CAT GAA GGG GCG T
cy5-hWt1-Right-3	TGA AGA CAC CGT GCG TGT GTT TAT ACG TCG AGT TGA ACG TCG TAA CA
cy5-hWt1-left-3	TAG CGC TAA CAA CTT ACG TCG TTA TGA TTC TGT ATT GGG CTC CGC A
cy5-hWt1-Right-4	AGG ATG GGC GTT GTG TGG TTT TAT ACG TCG AGT TGA ACG TCG TAA CA
cy5-hWt1-left-4	TAG CGC TAA CAA CTT ACG TCG TTA TGA TCG CTC TCG TAC CCT GTG C
cy5-hWt1-Right-5	GTG CGA GGG CGT GTG ACC GTT TAT ACG TCG AGT TGA ACG TCG TAA CA
cy5-hWt1-left-5	TAG CGC TAA CAA CTT ACG TCG TTA TGA GCT GGG CGT CCC GTC GAA G
cy3-hTnni3-Right-1	CTC CGC AGG GTG GGC CGC TTT TAT ACG TCG AGT TGA CCG ACG TAT TG
cy3-hTnni3-left-1	TAT TCG TTC GAA CTT ACG TCG TTA TGA AAC TTG CCT CGA AGG TCA A
cy3-hTnni3-Right-2	GAC TTT TGC CTC TAT GTC GTT TAT ACG TCG AGT TGA CCG ACG TAT TG
cy3-hTnni3-left-2	TAT TCG TTC GAA CTT ACG TCG TTA TGA TCT CTC TTC ATC CAC CTT G
cy3-hTnni3-Right-3	GGC GTG CGG CTC CGT GGC ATT TAT ACG TCG AGT TGA CCG ACG TAT TG
cy3-hTnni3-left-3	TAT TCG TTC GAA CTT ACG TCG TTA TGA AGC GCG GTA GTT GGA GGA G
cy3-hTnni3-Right-4	GGT GCA GGG CGA GGT TCC CTT TAT ACG TCG AGT TGA CCG ACG TAT TG
cy3-hTnni3-left-4	TAT TCG TTC GAA CTT ACG TCG TTA TGA GCC GCA TCG CTG CTC CCA T
cy3-hTnni3-Right-5	CAA ACT TTT TCT TGC GGC CCT TAT ACG TCG AGT TGA CCG ACG TAT TG
cy3-hTnni3-left-5	TAT TCG TTC GAA CTT ACG TCG TTA TGT CCA TTC CAC TCA GTG CAT C

Supplementary Table 5 The H probes used for single molecular in situ hybridization (PLISH).
