# Supplementary material for: Computational profiling of hiPSC-derived heart organoids reveals chamber defects associated with NKX2-5 deficiency
Source: Commun Biol. 2022 Apr 29;5:399. doi: 10.1038/s42003-022-03346-4 (PMC9054831; doi:10.1038/s42003-022-03346-4)
Supplement: Supplementary file 2 — Description of Additional Supplementary Files [file 42003_2022_3346_MOESM2_ESM.pdf]

Description of Additional Supplementary Files

File name: Supplementary Data 1

Description: Inter-cluster differentially expressed genes (DEGs) depicted in Fig. 2cii (fdr < 0.001). At most, twenty top DEGs are reported for each cluster. The column labeled ‘cluster’ indicates which cluster the gene is up regulated in.

File name: Supplementary Data 2

Description: Inter-cluster differentially expressed genes depicted in Fig. 4bii (fdr < 0.001). At most, twenty top DEGs are reported for each cluster. The column labeled ‘cluster’ indicates which cluster the gene is up regulated in.

File name: Supplementary Data 3

Description: Inter-cluster differentially expressed genes depicted in Supplementary Fig. 8bii (fdr < 0.1). At most, twenty top DEGs are reported for each cluster. The column labeled ‘cluster’ indicates which cluster the gene is up regulated in.

File name: Supplementary Data 4

Description: Differentially expressed genes between stages and differentiation methods for each cardiac cell type depicted in Supplementary Fig. 11ei-iii (fdr < 0.001). The column labeled ‘cell_type’ indicates which cell type was used to calculate the DEGs, while the column labeled ‘stage_and_differentiation_method’ indicates which group (Day 15 Org, Day 15 ML, Day 30 Org, or Day 30 ML) the gene is up regulated in.

File name: Supplementary Data 5

Description: Differentially expressed genes between wild type and mutant cells for each cardiac cell type depicted in Fig. 9f (CMs) and Supplementary Fig. 14ai (FBs), and Supplementary Fig. 14bi (Endo_ECs) (fdr < 0.001). The column labeled ‘cell_type’ indicates which cell type was used to calculate the DEGs, while the column labeled ‘WTvsMUT’ indicates which group (wild type vs mutant cells) the gene is up regulated in.

File name: Supplementary Data 6

Description: Cell type annotations used for the training set, Cui et al.

File name: Supplementary Data 7

Description: Cell type annotations used for the development set 1 from Asp et al.

File name: Supplementary Data 8

Description: Cell type annotations used for the development set 2 from Miao et al.

File name: Supplementary Data 9

Description: Important genes selected by the first random forest model and used as features in the second random forest model. The column labeled ‘classifier’ indicates which type of classifier (cell type, zone, or laterality) was being trained, while the column labeled ‘dataset’ indicates which test set (cross validation, development set 1, development set 2, wild type unsorted hiPSC, mutant unsorted hiPSC, or sorted hiPSC data) was being predicted on when the important genes were selected. For cross validation, the intersection of important genes for each iteration is reported.

File name: Supplementary Data 10

Description: Spreadsheet containing source data used to generate main figures.

File name: Supplementary Video 1

Description: Beating RA- heart organoids at day 30 from WTC line with an ACTN2-eGFP reporter.

File name: Supplementary Video 2

Description: Beating RA+ heart organoids at day 30 from WTC line with an ACTN2-eGFP reporter.

File name: Supplementary Video 3

Description: Beating RA- heart organoids at day 15 from an isogeneic line carrying NKX2-5 c.673C>A variant (PM28).

File name: Supplementary Video 4

Description: Beating RA+ heart organoids at day 15 from an isogeneic line carrying NKX2-5 c.673C>A variant (PM28).

File name: Supplementary Video 5

Description: Beating RA- heart organoids at day 15 from the WTC with the first exon deletion of NKX2-5 (Del33).

File name: Supplementary Video 6
Description: Beating RA+ heart organoids at day 15 from the WTC with the first exon deletion of NKX2-5 (Del33).
