# Supplementary material for: Computational profiling of hiPSC-derived heart organoids reveals chamber defects associated with NKX2-5 deficiency
Source: Commun Biol. 2022 Apr 29;5:399. doi: 10.1038/s42003-022-03346-4 (PMC9054831; doi:10.1038/s42003-022-03346-4)
Supplement: Supplementary file 19 — Reporting Summary [file 42003_2022_3346_MOESM19_ESM.pdf]

nature research

Corresponding author(s): Guang Li, Dennis Kostka

Last updated by author(s): Oct 28, 2021

Reporting Summary

Nature Research wishes to improve the reproducibility of the work that we publish. This form provides structure for consistency and transparency in reporting. For further information on Nature Research policies, see our Editorial Policies and the Editorial Policy Checklist.

Statistics

For all statistical analyses, confirm that the following items are present in the figure legend, table legend, main text, or Methods section.

n/a	Confirmed	
☐	☒	The exact sample size (n) for each experimental group/condition, given as a discrete number and unit of measurement
☐	☒	A statement on whether measurements were taken from distinct samples or whether the same sample was measured repeatedly
☐	☒	The statistical test(s) used AND whether they are one- or two-sided<br>*Only common tests should be described solely by name; describe more complex techniques in the Methods section.*
☐	☒	A description of all covariates tested
☐	☒	A description of any assumptions or corrections, such as tests of normality and adjustment for multiple comparisons
☐	☒	A full description of the statistical parameters including central tendency (e.g. means) or other basic estimates (e.g. regression coefficient) AND variation (e.g. standard deviation) or associated estimates of uncertainty (e.g. confidence intervals)
☒	☐	For null hypothesis testing, the test statistic (e.g. F, t, r) with confidence intervals, effect sizes, degrees of freedom and P value noted<br>*Give P values as exact values whenever suitable.*
☒	☐	For Bayesian analysis, information on the choice of priors and Markov chain Monte Carlo settings
☐	☒	For hierarchical and complex designs, identification of the appropriate level for tests and full reporting of outcomes
☒	☐	Estimates of effect sizes (e.g. Cohen's d, Pearson's r), indicating how they were calculated

Our web collection on statistics for biologists contains articles on many of the points above.

Software and code

Policy information about availability of computer code

Data collection	10X Genomics Chromium 120223; Chromium Single cell 3' GEM, Library & Gel Bead kit V3 PN-1000092; Illumina HiSeq X;
Data analysis	Cell Ranger; R packages: deMULTIplex, scds, scran, scater, igraph, ranger

For manuscripts utilizing custom algorithms or software that are central to the research but not yet described in published literature, software must be made available to editors and reviewers. We strongly encourage code deposition in a community repository (e.g. GitHub). See the Nature Research guidelines for submitting code & software for further information.

Data

Policy information about availability of data

All manuscripts must include a data availability statement. This statement should provide the following information, where applicable:

- Accession codes, unique identifiers, or web links for publicly available datasets
- A list of figures that have associated raw data
- A description of any restrictions on data availability

Sequencing data is deposited on GEO: GSE163619; reviewer access token: ofejsoqedpypbqj

Field-specific reporting

Life sciences study design

All studies must disclose on these points even when the disclosure is negative.

Sample size	32,458 scRNA-seq profiles of cells overall were analyzed
Data exclusions	Low quality cells, MULTI-seq doublets and negatives and computationally annotated multiplets were filtered out
Replication	Two biological replicates for the wild type scRNA-seq data. There is only one replicate for the mutant scRNA-seq data, but two single-variant mutant clones were profiled and one first exon deletion.
Randomization	none
Blinding	none

Reporting for specific materials, systems and methods

We require information from authors about some types of materials, experimental systems and methods used in many studies. Here, indicate whether each material, system or method listed is relevant to your study. If you are not sure if a list item applies to your research, read the appropriate section before selecting a response.

Materials & experimental systems

n/a	Involved in the study
☐	☒ Antibodies
☐	☒ Eukaryotic cell lines
☒	☐ Palaeontology and archaeology
☒	☐ Animals and other organisms
☒	☐ Human research participants
☒	☐ Clinical data
☒	☐ Dual use research of concern

Methods

n/a	Involved in the study
☒	☐ ChIP-seq
☐	☒ Flow cytometry
☒	☐ MRI-based neuroimaging

Antibodies

Antibodies used: cTNT, CDH5, Myl7, Nr2f2, MYH6, MYH7, ID2, HEY2, COL1A1, NFATC1, MYH11 antibodies were used

Validation:

Anti-Cardiac Troponin T Antibody (13-11) purchased from Invitrogen Catalog # MA5-12960,monoclonal mouse/IgG1. Species validated in dog, rat, hamster, zebrafish, mouse, human.45 Publications validated for Immunocytochemistry (ICC),7 Publications validated for Immunofluorescence (IF).Expression of Cardiac Troponin T was observed specifically in heart tissue and was negative for skeletal muscle and lung tissue in western blot.

Anti-CDH5 (D87F2) XP® antibody purchased from Cell Signaling #2500, monoclonal Rabbit/IgG. Species validated in human, mouse, rat,hamster,monkey,mink,chicken,D. melanogaster,Xenopus,zebrafish,Bovine,dog,pig,S. cerevisiae,C. elegans and horse.Suitable for western blot, immunoprecipitation,immunohistochemistry,chromatin immunoprecipitation, immunofluorescence,flow cytometry and ELISA-Peptide.101 citations provided on the manufacturer's website.

Anti-MYL7 antibody purchased from Sigma-Aldrich SAB2701294, polyclonal rabbit/IgG. Species validated in rat, mouse, human. Suitable for immunohistochemistry and western blot. Staining validated in iPS cell-derived cardiogenicity.

Anti-human COUP-TF II/NR2F2 Antibody purchased from R&D systemsCatalog Number: PP-H7147-00,monoclonal mouse / IgG2A. Species validated in mouse and human. Publications validated for western blot,direct ELISA,immunoprecipitation and immunohistochemistry. This antibody specifically recognizes human COUP-TF II and cross-reacts with mouse and rat COUP-TF II, but not cross-react with human COUP-TF I or EAR2.

Anti-MYH6 purchased from DSHB, S46, monoclonal mouse IgG1.Positive Tested Species Reactivity: Chicken, Human, Mouse, Quail, Rat, Zebrafish. Recommended Applications: Immunofluorescence, Immunohistochemistry, Western Blot.

Anti- MYH7 purchased from DSHB, BA-D5, monoclonal mouse IgG2b.Positive Tested Species Reactivity: Bovine, Canine, Fish, Goat, Guinea Pig, Horse, Human, Lamb, Llama, Mouse, Porcine, Rabbit, Rat, Zebrafish.Recommended Applications: Immunofluorescence, Immunohistochemistry, Western Blot.

Anti- ID2 purchased from DSHB, PCRP-ID2-1A8, monoclonal mouse IgG2b. Predicted Species Reactivity: Chick, Human, Xenopus. Recommended Applications: Immunoprecipitation, Microarray.

Anti- HEY2 purchased from DSHB, PCRP-HEY2-1H10, monoclonal mouse IgG2c. Predicted Species Reactivity: Human, Mouse. Recommended Applications: Immunoprecipitation, Microarray.

Anti- COL1A1 purchased from DSHB, SP1.D8, monoclonal mouse IgG1. Positive Tested Species Reactivity: Avian, Bovine, Canine, Chicken, Chondrichthyes, Human, Mouse, Porcine, Rabbit, Rat, Sheep, Turtle, Xenopus, Zebrafish. Recommended Applications: ELISA, FFPE, Immunofluorescence, Immunohistochemistry, Western Blot.

Anti-NFATC1 purchased from DSHB, PCRP-NFATC1-1A2, monoclonal mouse IgG1. Positive Tested Species Reactivity: Human. Recommended Applications: ELISA, Immunoprecipitation.

Anti-Cardiac Troponin T purchased from Abcom, ab45932, rabbit polyclonal IgG.Tested applications for: IHC-P, Sandwich ELISA, WB. Reacts with: Human. Predicted to work with: Rat, Rabbit, Guinea pig, Cow, Cat, Dog.

Anti-Myosin, Smooth Muscle Heavy Chain/MYH11 purchased from Biomedical Technologies, BT-562, rabbit IgG. Reactivity :human, mouse, rat, zebrafish. Application :western blot, immunohistochemistry, immunocytochemistry, immunohistochemistry - paraffin section, immunohistochemistry - frozen section.

Eukaryotic cell lines

Policy information about cell lines

Cell line source(s)	Allen Cell Collection and Stanford Cardiovascular Institute
Authentication	MTAs were approved for both cell lines
Mycoplasma contamination	none
Commonly misidentified lines (See ICLAC register)	*Name any commonly misidentified cell lines used in the study and provide a rationale for their use.*

Flow Cytometry

Plots

Confirm that:

- [x] The axis labels state the marker and fluorochrome used (e.g. CD4-FITC).
- [x] The axis scales are clearly visible. Include numbers along axes only for bottom left plot of group (a 'group' is an analysis of identical markers).
- [x] All plots are contour plots with outliers or pseudocolor plots.
- [x] A numerical value for number of cells or percentage (with statistics) is provided.

Methodology

Sample preparation	The heart organoid and monolayer derived cells were dissociated with collagenase and trypsin, respectively. After multiple times of washing with PBS, the dissociated cells were resuspended in PBS and filtered through a BD blue cap tube.
Instrument	BD Ariall
Software	Flowjo v10.6.1
Cell population abundance	1 million cells in each plot
Gating strategy	We used a hiPSC line without GFP signal as negative control to guide the gating.

- [ ] Tick this box to confirm that a figure exemplifying the gating strategy is provided in the Supplementary Information.
